# An acyclic nucleoside phosphonate effectively blocks the egress of the malaria parasite by inhibiting the synthesis of cyclic GMP

**DOI:** 10.1126/sciadv.ady2859

**Published:** 2025-11-21

**Authors:** Marie Ali, Rea Dura, Marc-Antoine Guery, Emma Colard-Itté, Thomas Cheviet, Léa Robresco, Laurence Berry, Corinne Lionne, Catherine Lavazec, Antoine Claessens, Suzanne Peyrottes, Kai Wengelnik, Sharon Wein, Rachel Cerdan

**Affiliations:** ^1^ LPHI, University of Montpellier, CNRS, Inserm, 34095 Montpellier, France.; ^2^ Institut Cochin, Université Paris Cité, CNRS, Inserm, 75014 Paris, France.; ^3^ IBMM, University of Montpellier, CNRS, ENSCM, 34293 Montpellier, France.; ^4^ CBS, University of Montpellier, CNRS, INSERM, 34090 Montpellier, France.

## Abstract

The urgent need for original antimalarial therapies arises from the alarming spread of malaria parasite resistance to existing drugs. A promising candidate, UA2239, an acyclic nucleoside phosphonate with a guanine as nucleobase, demonstrates rapid and irreversible inhibitory effects on *Plasmodium* parasites. It blocks the active exit process, named egress, of merozoites and gametes from infected erythrocytes. UA2239 disrupts the essential cyclic guanosine monophosphate (cGMP)–dependent egress pathway by decreasing cGMP levels in the parasite, strongly suggesting *Plasmodium falciparum* guanylyl cyclase α as its primary target. We also uncovered remarkable molecular mechanisms of resistance developed by parasites after prolonged exposure to the drug, which involve mutating not the target itself, but downstream effectors. The unique mechanism of action of UA2239 makes it a valuable first-in-class candidate for further development. Its ability to inhibit both parasite growth and transmission highlights its therapeutic potential as a dual-stage antimalarial agent.

## Teaser

An acyclic nucleoside phosphonate traps malaria parasites inside red blood cells by reducing cyclic GMP levels.

## INTRODUCTION

Malaria is caused by parasites of the genus *Plasmodium* with most fatal cases occurring after *Plasmodium falciparum* infection. Despite the effort and progress made in recent decades to control the disease (*1*), the number of deaths due to malaria was still estimated at 597,000 in 2023. After a notable increase at the beginning of the COVID-19 pandemic, malaria cases and deaths have been falling slowly for the past 3 years but remain higher than before the pandemic. The very first vaccines are currently administered in a selected number of African countries (*1*, *2*) and other vaccine candidates are under development (*3*, *4*). Nevertheless, chemotherapy remains indispensable to treat the estimated 263 million cases every year (*1*). Current treatments are based on artemisinin combination therapy (*1*). The emergence of partial resistance to artemisinin in Asia (*5*) and more recently in Africa (*1*), in addition to already established resistance to all commercially available drugs, worsens the situation and emphasizes the urgency of finding alternative options. Effective treatments are therefore necessary to bolster the therapeutic arsenal. The development of new chemical entities with novel mechanisms of action is a priority to face the increasing resistance of parasites to available medicines. Future antimalarial treatments are expected to target at least two stages of the complex life cycle of the parasite, in the human host or during the transmission by mosquitoes (*6*). In humans, *P. falciparum* multiplies initially in liver cells and after release of merozoites into the blood stream, replicates asexually in red blood cells (RBCs) in 48-hour cycles of invasion-growth-multiplication. Within the RBC, the parasite’s asexual cycle progresses through three stages: ring, trophozoite, and schizont. This culminates in the formation of up to 32 daughter cells (merozoites), which actively exit the host cell—a process called egress—to invade new RBCs (*7*). A fraction of parasites differentiates into transmissible sexual forms (male and female gametocytes) by passing through five morphological stages (stages I to V) in over 10 to 12 days. After a blood meal, male and female gametocytes ultimately egress and mate in the mosquito gut (*8*). Following several weeks of growth and multiplication, parasites settle in the salivary glands ready to be injected in a human host.

**Fig. 1.**
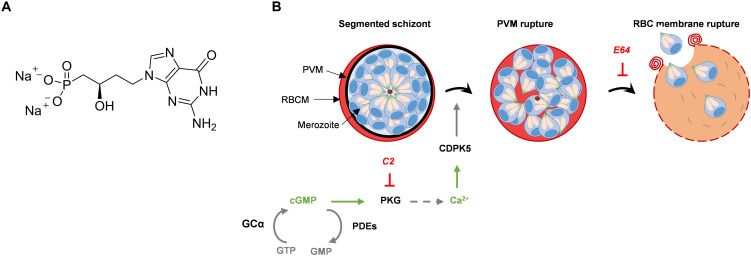
Chemical structure of UA2239 (A) and schematic representation of merozoite egress and the signaling cascades (B). PVM, parasitophorous vacuole membrane; RBC, red blood cell; RBCM, RBC membrane; PKG, cGMP-dependent protein kinase; GCα, guanylyl cyclase alpha; PDEs, phosphodiesterases; CDPK5, calcium-dependent protein kinase 5. Inhibitions by C2 and E64 are indicated in red.

We recently described a previously unexplored series of nucleotide analogs from the acyclic nucleoside phosphonate (ANP) family, which exhibit remarkable antimalarial activity (*9*). The best activities were observed with the chemical series of purine analogs (*9*). The lead compound UA2239 (Fig. 1A), a guanosine monophosphate (GMP) analog has strong activity in vitro on *P. falciparum* with a concentration to inhibit 50% of the parasite growth (IC_50_) of 74 nM for the 3D7 strain and of 30 and 45 nM for the chloroquine (CQ)–resistant strains FcM29 and W2, respectively. UA2239 is also active in vivo in *Plasmodium berghei*–infected mice with a low efficient dose to inhibit 50% of the parasite growth (ED_50_) of 0.5 mg/kg after intraperitoneal administration (*9*). UA2239 has no activity on mammalian K562 cells resulting in a very high selectivity index (SI > 10,000) (*9*). Nucleoside and nucleotide analogs are widely used as therapeutic agents in the clinical treatment of viral infections and of cancer due to their antiproliferative properties (*10*, *11*). Their mechanism of action generally involves their conversion into the corresponding poly-phosphorylated derivatives and their interaction with cellular or viral polymerases (as substrates and/or competitive inhibitors) (*10*). Unexpectedly, initial phenotypic characterization of the mode of action of UA2239 on *P. falciparum* revealed no effect on parasite intraerythrocytic development including the formation of merozoites. This contradicts the hypothesis of inhibiting DNA synthesis. The absence of newly formed ring stages, coupled with the persistent accumulation of schizonts, supports impaired egress as the underlying mechanism of action (*9*). In *Plasmodium*, both merozoites and gametocytes exit from the RBC in a tightly controlled manner involving several layers of regulation (*12*, *13*). A few minutes before rupture of the parasitophorous vacuole membrane (PVM), accumulation of the intracellular messenger cyclic GMP (cGMP) activates cGMP-dependent protein kinase (PKG) that in turn triggers a calcium signal essential for egress progression (Fig. 1B) (*14*–*16*). Cellular cGMP levels are tightly regulated by a balance between its synthesis from guanosine 5′-triphosphate (GTP) by guanylyl cyclases (GC) (*17*) and its hydrolysis to GMP by phosphodiesterases (PDEs) (Fig. 1B) (*18*). Specific inhibitors of *Pf*PKG have been developed and lead to impairment of egress with the fully segmented parasites being blocked within the RBC. Compound 2 (C2, also known as ML1, an imidazopyridine derivative) targets the catalytic domain of *Pf*PKG and a medicinal chemistry program led to the development of ML10, a more potent C2 analog (*19*–*21*). C2 blocks parasite egress reversibly (*14*) and is therefore widely used in *Plasmodium* cell biology. Another compound belonging to the trisubstituted imidazole family also inhibits *Pf*PKG and exhibits a potent activity against liver stages, as well as asexual and sexual blood stages of the parasite (*22*). In contrast, inhibitors of *Pf*PDEs that increase cellular levels of cGMP induce premature egress of merozoites, impairing the invasion of new RBC (*23*–*26*). Among this trio of enzymes, PDE, GC and PKG, the only enzyme for which no inhibitor has yet been developed is GC despite the importance of the cGMP-dependent cellular processes for parasite survival. The *Plasmodium* genome encodes two GC proteins. The GCα is essential at the blood stage and for gametogenesis (*17*) and GCβ is required at the mosquito stage for ookinete development (*27*).

In this work, we characterize the pharmacological properties of UA2239 and show that it has activity against the asexual blood stages and the transmission stages of the parasite. The compound does not inhibit DNA synthesis but irreversibly blocks parasite egress. Its unique mode of action consists of disrupting the crucial cGMP-dependent egress pathway by reducing the level of cGMP in the parasite. We also reveal remarkable molecular mechanisms of resistance evolved by parasites in response to prolonged drug exposure.

## RESULTS

### UA2239 is active when applied on trophozoites or schizonts and enters iRBCs through NPPs

**Fig. 2.**
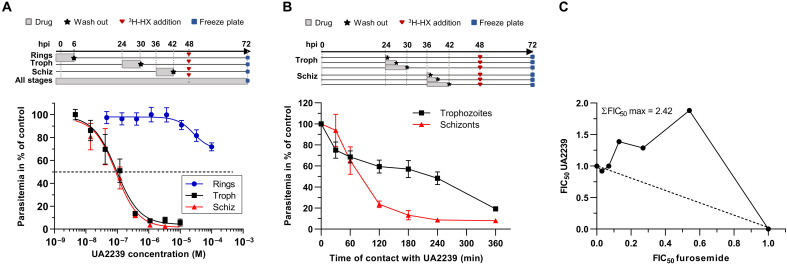
Activity of UA2239 on asexual blood stage parasites. (**A**) Activity of UA2239 on ring, trophozoite, and schizont stages after 6 hours of contact with various concentrations of UA2239 (*n* = 3, means ± SEM). The experimental setup is indicated at the top. Gray bars correspond to the time of drug contact. (**B**) Time course of *P. falciparum* growth inhibition by UA2239 depending on the parasite stage. Synchronous cultures at the trophozoite stage (24 hpi) or schizont stage (36 hpi) were treated with 750 nM UA2239 for the indicated time. Cells were then washed and resuspended in fresh media as shown in the scheme at the top (*n* = 3; means ± SEM). (**C**) Interaction between UA2239 and furosemide represented as an isobologram. Data presented are from one typical experiment (*n* = 3). The maximal sum of FICs of UA2239 and furosemide in combination (ΣFIC50 max) is indicated on the graph.

We analyzed whether susceptibility to UA2239 differs according to the stage of parasite development. For this, synchronous ring, trophozoite, or schizont cultures were exposed for 6 hours to UA2239, and parasite viability was quantified during the following cycle. The compound was not active when used at the ring stage (Fig. 2A). However, a treatment during trophozoite or schizont development resulted in an IC_50_ of 178 and 143 nM, respectively. These values are close to the IC_50_ of 74 nM found when parasites are treated for 48 hours. To determine the minimum contact time with the drug required to observe a significant effect, synchronized cultures at the trophozoite or schizont stage were treated during a short pulse (from 0.5 to 6 hours) with 750 nM UA2239 (~10 × IC_50_) (Fig. 2B). For schizonts, a 2-hour contact with the compound was sufficient to kill 75% of the parasites and an additional hour of incubation resulted in 90% inhibition of parasite growth. For trophozoites, a 6-hour contact was required to achieve 75% growth inhibition (Fig. 2B). UA2239 thus exerts a rapid and irreversible cytotoxic effect when added at the trophozoite or schizont stage. One common chemical attribute of all ANPs is their anionic character that limits their passive diffusion across biological membranes. We thus explored the mechanism of entry of UA2239 into the infected RBC (iRBC). *Plasmodium* relies on specific channels in the iRBC membrane known as new permeation pathways (NPPs) to facilitate the uptake of molecules of low molecular weight such as carbohydrates, nucleosides, nucleobases, ions, etc. (*28*). These channels are induced by the parasite and are active in trophozoites and schizonts. To test whether UA2239 enters the iRBC via NPPs, we quantified the effect of furosemide, a potent inhibitor of the NPPs (*29*), on UA2239 activity. The isobologram method allows to determine whether two compounds in combination exert antagonistic, additive, or synergistic effects (*30*). The maximal sum of the fractions of IC_50_ (ΣFIC_50_ max) was found to be 2.25 ± 0.12 (means ± SEM, *n* = 3) demonstrating that furosemide reduces the antimalarial activity of UA2239 and thus acts as an antagonist (Fig. 2C). This result indicates that UA2239 enters the iRBC through NPPs. This could explain the lack of UA2239 activity when applied to ring-stage parasites, as NPPs are not yet established at this stage, thereby preventing drug entry into the iRBC.

### UA2239-treated parasites are blocked at the egress step

**Fig. 3.**
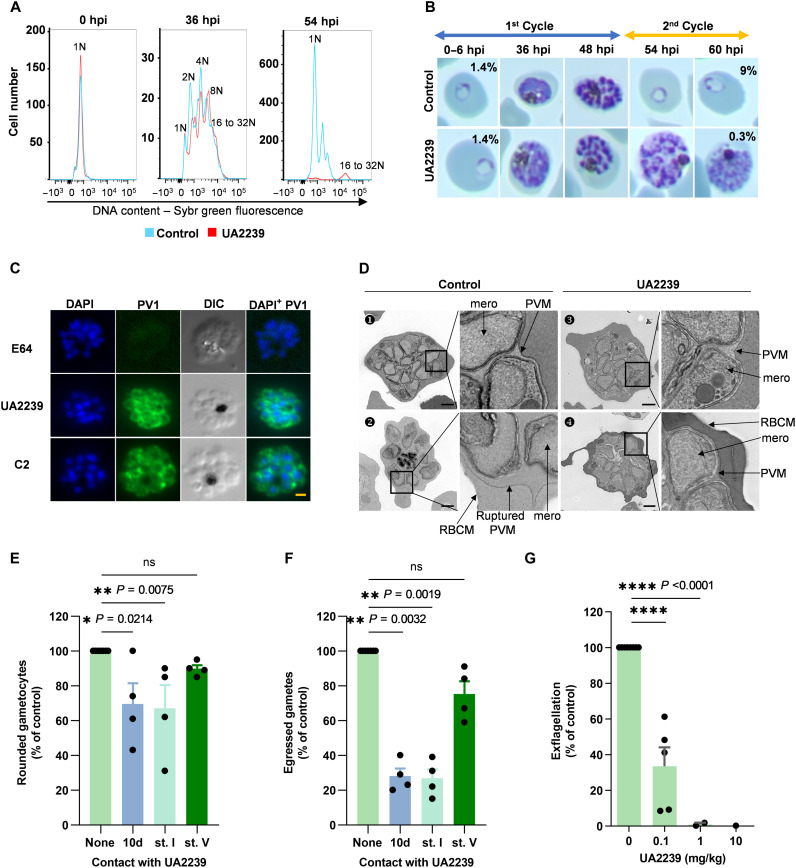
UA2239 inhibits the egress step in asexual blood stage parasites and gametocytes. (**A**) Effect of UA2239 on DNA content determined by flow cytometry. N represents the genome equivalents. (**B**) Effect of UA2239 on parasite development followed by colored thin blood smears. Parasitemia is indicated for 6 and 60 hpi. Data are from one of two independent experiments. For (A) and (B), UA2239 concentration was 2 μM. (**C**) IFA on PV1-GFP *P. falciparum* parasites. Late schizont stage parasites were incubated for 4 hours with UA2239, C2, or E64. The intact PVM is visualized by immunofluorescence detection of PV1-GFP. Nuclei were stained with Hoechst (blue). Scale bar, 1 μm. (*n* = 3). (**D**) Electron microscopy images of control and UA2239-treated RBCs infected with segmented schizonts. The right panels are close-up views of the framed area on the left panels. (1) Untreated iRBC with intact PVM, (2) untreated iRBC with a ruptured PVM, (3) iRBCs treated with UA2239 from 6 to 44 to 46 hpi, and (4) iRBCs treated with UA2239 from 36 hpi to 44 to 46 hpi. mero, merozoite. Scale bar, 1 μm. (**E** and **F**) Effect of UA2239 (10 × IC_50_) on *P. falciparum* gametogenesis. Gametocytes were treated or not with UA2239 for 10 days (10d) (from stage I to stage V), for 24 hours at stage I (st. I) or for 24 h at stage V (st. V). On day 10, rounding-up (E) and gamete egress (F) were quantified relative to untreated controls (*n* = 4, means ± SEM). A Kruskal-Wallis nonparametric test was used to calculate *P* values (ns, not significant). (**G**) Effect of UA2239 on *P. berghei* gametogenesis after intraperitoneal treatment. No exflagellation center was detected after treatment with 10 mg/kg. (*n* = 2 to 5, means ± SD). Two-tailed Student’s *t* test was used to calculate *P* value (*P* < 0.0001).

Since ANPs are described as inhibitors of DNA synthesis (*10*, *31*–*34*), we initially analyzed the effect of UA2239 treatment on parasite DNA content by flow cytometry. For both the control and the treated cultures, the DNA content increased similarly over time from 1 copy (1 N) up to 16 to 32 N showing that DNA synthesis was not affected (Fig. 3A). UA2239-treated parasites developed normally until the late schizont segmenter stage when merozoites are formed but parasites failed to progress to the next cycle (Fig. 3B). Merozoite egress is a well-characterized process in which the PVM ruptures before lysis of the RBC membrane (Fig. 1B). To identify the specific blockade point in UA2239-treated parasites, we assessed PVM integrity by fluorescence microscopy using a transgenic *P. falciparum* 3D7 strain expressing green fluorescent protein (GFP)–tagged parasitophorous vacuolar protein 1 (*Pf*PV1) (Fig. 3C) (*35*). In case of PVM rupture, the PV1-GFP fluorescence diffuses into the RBC cytosol. As controls, we used the *Pf*PKG inhibitor C2 and the protease inhibitor E64 (*36*), which block egress before and after PVM rupture, respectively (Fig. 1B). UA2239 blocked PVM rupture similarly to C2 treatment, thus inhibiting an initial step of merozoite release (Fig. 3C). Last, we analyzed the ultrastructure of UA2239-treated parasites by electron microscopy. Treatment was started either at 6 or 36 hours postinvasion (hpi), and parasites were imaged at 44 to 46 hpi (Fig. 3D). Eighty-nine and 98% of the segmented iRBCs showed an intact PVM after treatment at 6 or 36 hpi, respectively, similar to what we observed for C2 (82%) (table S1). In addition, the distinct contrast between the RBC cytoplasm and the parasitophorous vacuole lumen suggests that the PVM was neither porated nor ruptured in the presence of UA2239 (Fig. 3D, images 3 and 4). In conclusion, UA2239 enters iRBCs through NPPs at the trophozoite and schizont stages but exerts its inhibitory effect later at the egress step.

### UA2239 inhibits gametogenesis of *P. falciparum* and *P. berghei*

We investigated whether UA2239 affects the ability of *Plasmodium* parasites to undergo sexual development and gametogenesis. UA2239 was added to synchronous *P. falciparum* gametocyte cultures either daily for 10 days from stage I to stage V, or only during 24 hours either at stage I or at stage V. UA2239 had no effect on gametocytemia, gametocyte morphology, and progression of gametocytes from stage I to V (fig. S1). Stage V gametocytes were then subjected to a gametogenesis assay scoring the ability of gametocytes to undergo rounding up and egress from the erythrocyte (*37*). UA2239 had slight but significant impact on gametocyte rounding up when the drug was added at day 1 for 24 hours (stage I) or when present over 10 days (Fig. 3E). The proportion of egressed gametes drastically decreased by approximately 70% (Fig. 3F). The effect was similar when gametocytes were treated for only 24 hours at stage I or for 10 days, consistent with the irreversible activity of UA2239. Rounding-up and egress were not affected when UA2239 was added at stage V. This result provides additional evidence that UA2239 enter the infected erythrocyte via NPPs, as NPPs are mainly active during the first 24 hours of gametocyte maturation (stage I) and then decline in later stages (*38*).

The effect of UA2239 was also determined ex vivo on male gametogenesis of the rodent parasite *P. berghei*. First, exflagellation was quantified in the blood of a *P. berghei*–infected mouse, and the value was used as positive control. Subsequently, the same mouse was treated with an intraperitoneal dose of UA2239, and exflagellation was again quantified 30 min after treatment. UA2239 reduced the exflagellation of male gametocytes in the mouse model, with an ED_50_ of 0.1 mg/kg (Fig. 3G). This dose is of the same order as the one observed against the asexual stage (*9*). In summary, our data show that UA2239 inhibits gametogenesis in *P. falciparum* and *P. berghei* at low doses. It is therefore likely that UA2239 blocks sexual reproduction in mosquitoes and hence inhibits parasite transmission.

### UA2239 resistant parasites carry mutations in *Pf*PKG and in *Pf*PDEβ

**Fig. 4.**
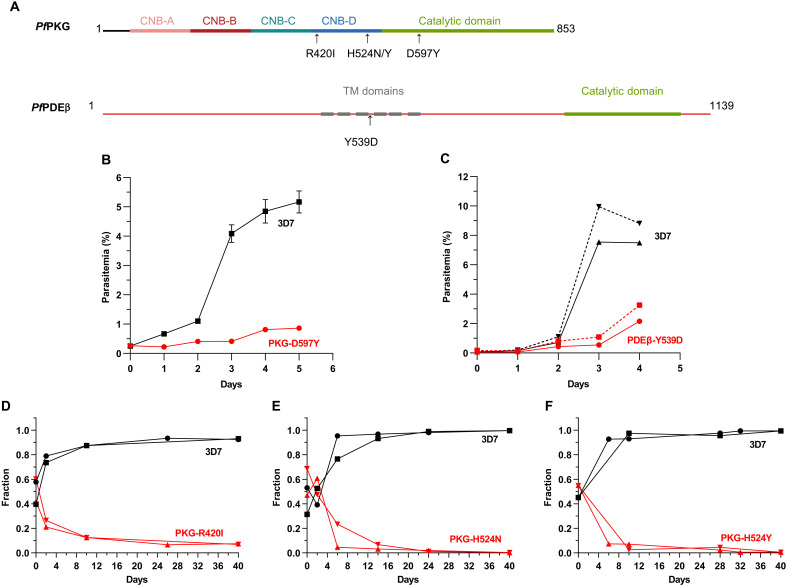
Mutations conferring resistance to UA2239 have a fitness cost for the parasite. (**A**) Schematic representation of the *Pf*PKG and *Pf*PDEβ domain organization. *Pf*PKG consists of four CNB domains designated CNB-A, CNB-B, CNB-C, and CNB-D, followed by a C-terminal catalytic domain. *Pf*PDEβ comprises six transmembrane (TM) domains and a C-terminal catalytic domain. The positions of the identified mutations are indicated. (**B**) Growth of PKG-D597Y GER-parasites was followed by stained thin blood smears over 6 days (*n* = 3). Some error bars are within the size of the symbols. (**C**) Growth of PDEβ-Y539D GER-parasites was followed by stained thin blood smears over 5 days. Two independent experiments starting at different initial parasitemia are shown. (**D** to **F**) Pairwise competitive growth assays with the 3D7 strain quantified by qPCR on genomic DNA for GER-parasites mutated in *Pf*PKG. (D) PKG-R420I, (E) PKG-H524N, and (F) PKG-H524Y. Cultures were started at comparable parasitemia levels for both strains, corresponding to a fraction of 0.5. The fraction increases to 1 for the faster-growing strain when it accounts for 100% of the parasite population (*n* = 2).

A common approach to identify the pharmacological target of a compound is to generate resistant parasites and then identify the genetic modifications leading to resistance. We used two different protocols to obtain UA2239-resistant 3D7 *P. falciparum* blood-stage parasites. Drug pressure was applied intermittently by drug-on/drug-off cycles either using the same concentration (10 × IC_50_) or gradually increasing the concentration of UA2239 (from 3 × IC_50_ to 10 × IC_50_). Parasites were cultured independently and in triplicates for 7 months. From all six independent and resistant populations, several clones were isolated. Two clones per population were selected for whole-genome sequencing (WGS). Their IC_50_ values for UA2239 increased by 35- to 111-fold compared to the parental 3D7 strain (74 nM). Focusing on protein-coding sequences, we found nonsynonymous mutations in 10 genes (fig. S2A). All clones except one had a single-nucleotide polymorphism (SNP) in the *Pfpkg* gene (PF3D7_1436600) resulting in four different and independent substitutions in the *Pf*PKG enzyme (R420I, H524Y, H524N, and D597Y) (fig. S2A). *Pf*PKG, the key regulator of merozoite egress, is an 853-residue protein comprising a C-terminal catalytic domain preceded by four cyclic nucleotide binding (CNB) domains. Among these, the CNB-A, CNB-B, and CNB-D domains are capable of binding cGMP (*16*, *39*, *40*). Three of the four mutations (R420I, H524N, and H524Y) are localized in the CNB-D domain; the fourth mutation (D597Y) is in the catalytic domain (Fig. 4A).

The second most prevalent mutated gene encodes gametocyte development protein 1 (GDV1, PF3D7_0935400). A stop codon within its reading frame was present in 8 of the 12 clones. GDV1 functions in gametocyte development (*41*, *42*) making it unlikely to be directly related to UA2239 resistance. The only clone without a mutation in *Pf*PKG has the nonsynonymous mutation Y539D in PDEβ (PF3D7_1321500) (fig. S2A). *Pf*PDE acts in the same signaling pathway as *Pf*PKG and negatively regulates the cellular levels of cGMP, the essential activator of *Pf*PKG. The Y539D resistance mutation is not localized in or close to the catalytic site but at the beginning of the fourth (of six) predicted transmembrane helices (Fig. 4A).

The analysis also revealed 11 microinsertions/deletions (INDELS) in the genomes of the resistant parasites (fig. S2B). None of the corresponding proteins is known to be related to parasite egress. Copy number variations were not present in the resistant clones.

For further analysis, we focused on the two mutated enzymes involved in the egress pathway. We selected one clone obtained after drug pressure (termed R-parasites) for each mutation in *Pf*PKG and for the unique mutation in *Pf*PDEβ (Table 1 and fig. S2A).

**Table 1.** Sensitivity of UA2239-resistant strains (R- and GER-parasites) to UA2239. Data are the means ± SD of three or four independent experiments performed in duplicates except for (*), which is the mean of six independent experiments.

Strain	IC_50_ of UA2239 (nM)	
WT 3D7	74.0 ± 16.2*	
	**R-parasites**	**GER-parasites**
*Pf*PKG - R420I	2 763 ± 662	568 ± 65
*Pf*PKG - H524N	4103 ± 1674	1340 ± 85
*Pf*PKG - H524Y	2908 ± 926	814 ± 152
*Pf*PKG - D597Y	3917 ± 334	693 ± 117
*Pf*PDEβ - Y539D	2592 ± 991	578 ± 163

### Single-site mutations of *Pf*PKG and *Pf*PDEβ confer resistance to UA2239

To confirm the involvement of the mutations in the resistance mechanisms, we introduced individually each identified mutation in the 3D7 strain using CRISPR-Cas9 (figs. S3 and S4). The five transgenic parasite lines were cloned, and genome editing was confirmed by polymerase chain reaction (PCR) and sequencing (figs. S3 and S4). All genome-edited resistant (GER) strains were found to be resistant to UA2239. The IC_50_ values increased 8- to 18-fold with respect to the 3D7 strain (Table 1) demonstrating that each mutation indeed confers resistance to UA2239. The GER-parasites showed lower IC_50_ values than the corresponding R-parasites (Table 1). It is likely that the 7 months of in vitro culture under drug pressure induced additional adaptations that cannot be detected by genome sequencing.

### Mutations conferring resistance to UA2239 have a fitness cost for the parasite

All GER-parasite clones showed a growth defect. For the PKG-D597Y and PDEβ-Y539D GER-parasites, lower parasitemia was easily observed by counting stained thin blood smears over 6 or 5 days, respectively. The multiplication rates of the PKG-D597Y and PDEβ-Y539D mutant parasites were approximately half that of the wild-type (WT) strain (Fig. 4, B and C). For the other PKG GER-parasites (R420I, H524N, or H524Y), the fitness cost was less drastic, allowing us to perform competitive growth assays. Equal numbers of synchronous 3D7 and GER-parasites were cocultured in over 20 cycles, and their relative abundance was determined by quantitative PCR (qPCR) on genomic DNA. The 3D7 strain outcompeted the genome-edited R420I, H524N, or H524Y strains quickly, in less than four cycles (Fig. 4, D to F). In conclusion, all mutations conferring resistance to UA2239 induce a heavy fitness cost for the parasite, resulting in a strong growth disadvantage compared to the WT strain.

### UA2239-resistant parasites show no cross resistance with standard antimalarial drugs

**Fig. 5.**
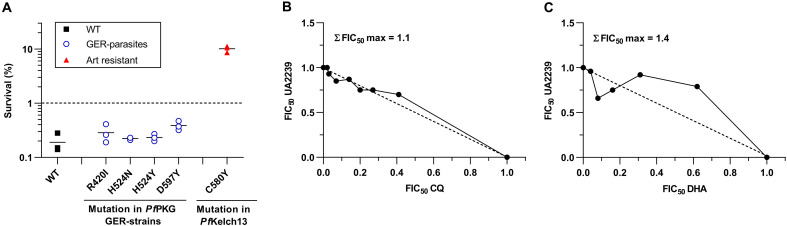
Interaction of UA2239 with other antimalarial drugs. (**A**) Sensitivity of UA2239-resistant strains to DHA. Sensitivity of GER-parasites to DHA was determined using RSA. A survival rate <1% (dashed line) indicates sensitive strains. 3D7 WT strain served as the sensitive control (black squares) and the NF54 Kelch13 C580Y line (red triangles) as resistant control (*74*) (*n* = 3 biological replicates). (**B** and **C**) In vitro interaction between UA2239 and CQ (B) or DHA (C) for their antimalarial activities against the 3D7 strain represented as an isobologram. Data presented are from one representative experiment (*n* = 3). The maximal ΣFIC_50_ (ΣFIC_50_ max) of the presented experiments are indicated on the graphs.

We determined the sensitivity of GER-parasites to the two standard antimalarial drugs, CQ and dihydroartemisinin (DHA). None of the *Pf*PKG or *Pf*PDEβ mutations induced a significant change in the IC_50_ value of CQ compared with the WT strain (fig. S5A). For artemisinin, a strain is considered resistant if its survival rate exceeds 1% in a classic ring-stage survival assay (RSA). For the PKG GER lines, survival rates were <1%, indicating that these mutations do not confer resistance to DHA (Fig. 5A). The same experiments were conducted with the R-parasites and showed similar results (fig. S5, A and B). Intriguingly, only the D597Y R-parasites had a survival rate of around 2% (fig. S5B), while the corresponding D597Y GER-parasites were sensitive to DHA, indicating that the D597Y mutation alone cannot be the cause of artemisinin resistance (Fig. 5A). The PDEβ mutated parasites were not tested for artemisinin resistance as the strain could not be properly synchronized in culture.

We also assessed the interactions between UA2239 and CQ or DHA at the pharmacological level using the isobologram method. We observed additive interactions in both combinations, with a mean ΣFIC_50_ max of 1.32 ± 0.15 and 1.34 ± 0.04 (±SEM) for CQ and DHA, respectively (Fig. 5, B and C). These results suggest that UA2239 could be used in association with either CQ or DHA.

### UA2239 inhibits the cGMP-PKG egress pathway through a novel mechanism of action

**Fig. 6.**
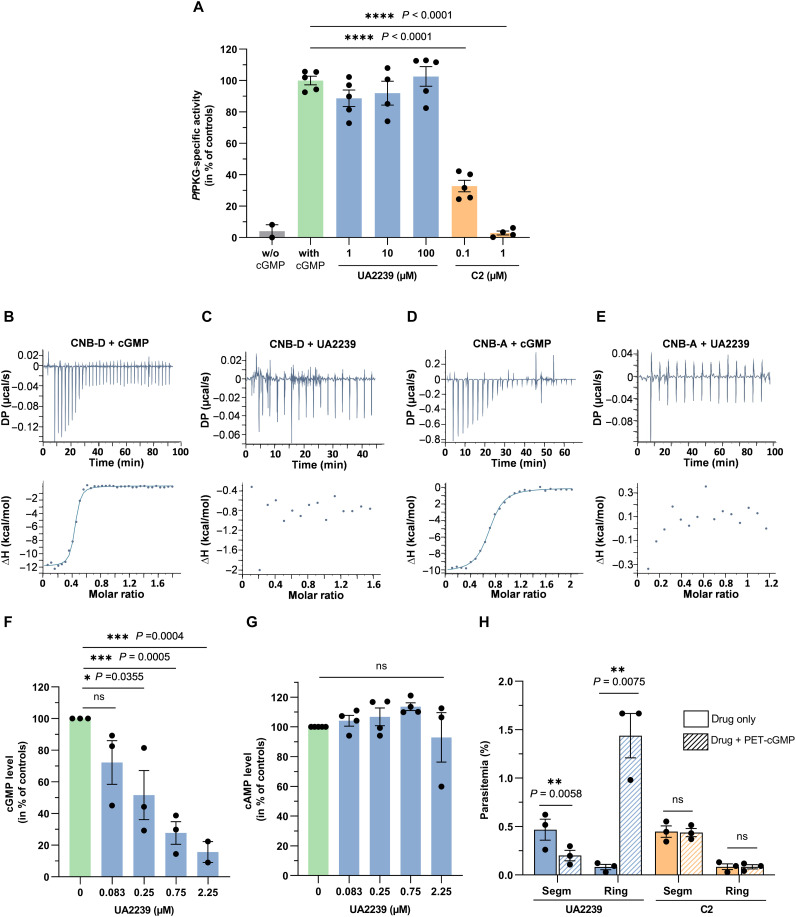
Mechanism of action of UA2239 on cGMP-dependent egress pathway. (**A**) Specific activity of the recombinant full-length *Pf*PKG was determined in presence of 10 μM cGMP or without (w/o) cGMP using ADP-Glo kinase kit. A final concentration of 50 nM *Pf*PKG was used. UA2239 was added at 1, 10, or 100 μM and C2 at 0.1 or 1 μM, *n* = 4 to 5 independent experiments (means ± SEM). For the condition in the absence of cGMP, *n* = 2 (means ± SD). Student’s *t* test was used to calculate *P* values (**** <0.0001). There was no significant difference between UA2239-treated (blue bars) and untreated control (green bar) samples. (**B** to **E**) ITC binding curves for the CNB-D and CNB-A constructs to cGMP or UA2239. Top and bottom panels display the ITC titration curves and the binding isotherms, respectively. The dissociation constant of cGMP for the CNB-D and CNB-A domains is 0.035 ± 0.005 and 1.03 ± 0.11 μM, respectively. UA2239 does not bind to the CNB-D or CNB-A domain. (**F**) Effect of UA2239 on intracellular cGMP levels (**G**) Effect of UA2239 on intracellular cAMP levels. For (F) and (G), purified 3D7 schizonts were allowed to develop in the presence of 1.5 μM C2 (to prevent egress) either without or with various concentrations of UA2239. Segmented schizonts were lysed with 0.1 M HCl and cGMP and cAMP levels were determined. Data are expressed in % of the untreated samples (*n* = 3 or 4 independent experiments, means ± SEM). Two-tailed Student’s *t* test was used to calculate *P* value. (**H**) Treatment with PET-cGMP reverses the UA2239-induced egress block, leading to next-generation ring-stages. Data are expressed as parasitemia for segmented (segm) or ring stage parasites. Ratio paired Students’s *t* test was used to calculate *P* value (ns, not significant).

To elucidate the mode of action of UA2239, we first aimed to determine whether PKG is its molecular target, as four independent PKG mutations were found to confer resistance. In addition, the effect of UA2239 on parasites resembles the one observed with the PKG inhibitor C2. UA2239 could impair *Pf*PKG activity, either by binding to the catalytic site, as C2 does, or by preventing the cGMP-induced activation. To rapidly test the first hypothesis, we took advantage of the availability of both UA2239-resistant and C2-resistant parasites (T618Q) (*43*) that we had previously generated (fig. S3). We assessed the sensitivity of the different R- and GER-parasites to C2 and inversely the sensitivity of the C2-resistant strain to UA2239. Our results revealed the absence of cross-resistance between UA2239 and C2 (Table 2) and an additive interaction between the two compounds (fig. S6). In parallel, we measured the activity of the recombinant full-length *Pf*PKG (provided by the Protein Biochemistry Platform of Geneva University) in the presence of UA2239 or C2 as a control. As expected, *Pf*PKG was activated by cGMP, and the activity was inhibited by C2. Unexpectedly, UA2239 did not impair *Pf*PKG activity, even at a concentration 10 times higher than that of cGMP (Fig. 6A). Last, we investigated the binding of UA2239 to two cGMP-binding domains of *Pf*PKG, using isothermal titration calorimetry (ITC). We produced and purified the high-affinity CNB-D domain and the low-affinity CNB-A domain in sufficient quantities for ITC analysis. CNB-D and CNB-A bound cGMP with a dissociation constant (*K*_d_) value of 0.035 ± 0.005 and 1.03 ± 0.11 μM, respectively (Fig. 6, B and D). These values are consistent with those reported in previous studies (*39*, *40*, *44*). However, no binding was detected between UA2239 and the two cGMP-binding domains (Fig. 6, C and E).

**Table 2.** Sensitivity of UA2239-resistant strains (R- and GER-parasites) to C2 and of C2-resistant strain (T618Q) to UA2239. Data are the means ± SD of three independent experiments performed in duplicates except for (*), which is the mean of six independent experiments.

Strain		Compound	IC_50_ (nM)	
WT 3D7		UA2239	74.0 ± 16.2*	
		C2	284.2 ± 48.2	
			**R-parasites**	**GER-parasites**
**UA2239-resistant**	*Pf*PKG -R420I	C2	271.4 ± 57.4	282.8 ± 60.3
	*Pf*PKG -H524N	C2	128.9 ± 56.8	167.4 ± 37.5
	*Pf*PKG -H524Y	C2	116.9 ± 7.1	167.4 ± 38.5
	*Pf*PKG -D597Y	C2	128.9 ± 59.9	274.2 ± 6.5
**C2-resistant**	*Pf*PKG -T618Q	UA2239	-	145.8 ± 37.4
	*Pf*PKG -T618Q	C2	-	3 511.7 ± 962.0

Together, these results show that UA2239 binds neither to the catalytic site nor to the CNB domains of the *Pf*PKG and therefore suggest a different primary target. Yet, several point mutations in *Pfpkg* confer resistance to UA2239, suggesting a functional link between the kinase and the mechanism of action of UA2239. This enzyme is activated by the cooperative binding of cGMP, with intracellular levels regulated by a balance between synthesis by GC and degradation by PDE. We therefore measured intracellular cGMP levels and observed that UA2239 treatment led to a dose-dependent reduction in cGMP content in segmented schizonts (Fig. 6F). The decrease could be attributed to the activation of PDEβ or the inhibition of GCα. PDEβ has been described to exhibit dual activity, hydrolyzing both cGMP and cAMP, and being the sole enzyme responsible for cAMP hydrolysis at the asexual stage (*25*). To assess the effect of UA2239 on PDEβ activity, we quantified the intracellular cAMP levels. UA2239 had no effect on cAMP levels (Fig. 6G), suggesting that PDE hydrolytic activity remains unaffected.

To further investigate the mechanism of action of UA2239, we artificially elevated cGMP levels by either inhibiting its hydrolysis by PDE or adding a membrane-permeable cGMP analog. We measured cGMP levels in the presence of the PDE inhibitor BIPPO (5-benzyl-3-isopropyl-1,6-dihydro-7H-pyrazolo [4,3-d]pyrimidin-7-one). The addition of BIPPO to UA2239-treated parasites resulted in increased cGMP levels (fig. S7). When UA2239-treated parasites were exposed to a membrane-permeable cGMP analog, ß-phenyl-1,N²-ethenoguanosine-3',5'-cyclic monophosphate (PET-cGMP) able to activate *Pf*PKG (*17*, *44*), we observed a significant decrease in the number of segmented parasites and an important increase in the numbers of rings (Fig. 6H) indicating a resumption of parasite growth. As expected, PET-cGMP did not complement C2-treated parasites (Fig. 6H). The effect of UA2239 can thus be partially reversed by impairing cGMP degradation or complemented by adding a cGMP analog.

Together, these results demonstrate that the mechanism of action of UA2239 is due to an insufficient level of cGMP in the parasites, which blocks the signaling cascade at the first step in the pathway (Fig. 1B). The inhibition of cGMP synthesis suggests that *Pf*GCα is the likely primary target.

### UA2239 is docked in the substrate/product-binding site of the *Pf*GCα 3D model

**Fig. 7.**
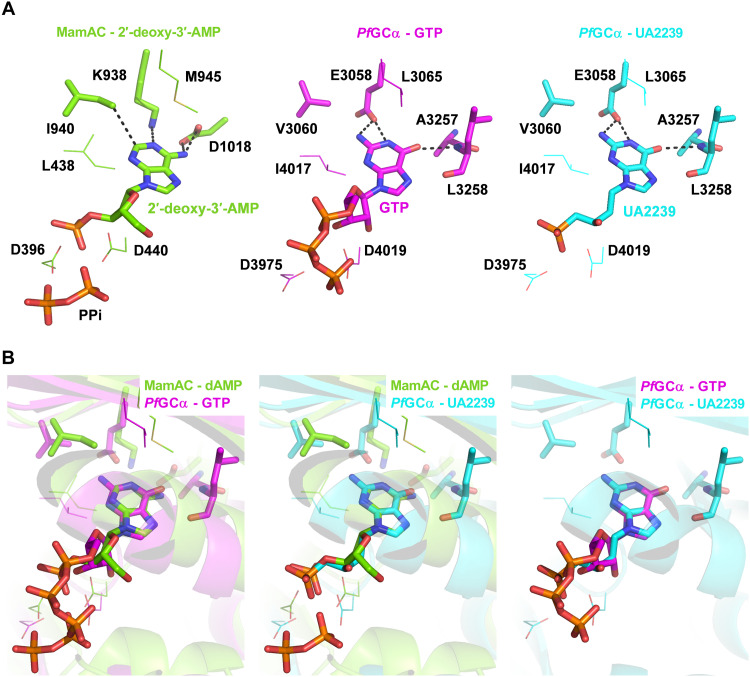
Docking of UA2239 into the substrate/product-binding site of the *Pf*GCα 3D model. (**A**) Binding pockets of MamAC in complex with 2′-deoxy-3′-AMP and PPi (PDB 1CS4) (left), of the *Pf*GCα model in complex with the substrate GTP (middle) or with UA2239 (right). (**B**) Overlays of MamAC (green) and *Pf*GCα (pink GTP ligand or blue UA2239 ligand) (left and middle) and overlay of *Pf*GCα with GTP or UA2239 (right). The ligands are shown as sticks. The residues sharing conserved interactions with the ligands in the two binding sites are shown as lines. The residues allowing the selective interactions with either an adenine or a guanine base are shown as sticks.

In the absence of a known three-dimensional (3D) structure, we modeled the GC domain of *Pf*GCα using AlphaFold2 (*45*) to predict whether UA2239 can bind to its active site. *Pf*GCα (PF3D7_1138400) is a bifunctional transmembrane enzyme of 4226 amino acids with an N-terminal P4–adenosine triphosphatase (ATPase)–like domain and a C-terminal GC domain. The GC domain consists of two catalytic subdomains, C1 and C2, each preceded by six transmembrane helices (*17*). The overall modeled structure is shown in fig. S8, where it is compared with the x-ray crystal structure of the cyclase domain of the mammalian adenylyl cyclase, MamAC [Protein Data Bank (PDB) 1CS4]. We chose this protein as a model anchor because, despite its relatively low sequence identity with GC domain of *Pf*GCα (26%), it shares one of the highest identities within the protein family. In addition, its structure is available at high resolution (<3 Å) in complex with a substrate analog, the 2′-deoxy-adenosine 3′-monophosphate (2′-deoxy-3′-AMP). The two overall structures align well with a root mean square deviation (RMSD) of 1.86 Å (fig. S8D). GTP, the substrate of *Pf*GCα, and UA2239 were docked into the AlphaFold2 model of the GC domain of *Pf*GCα using the 2′-deoxy-3′-AMP of MamAC as an anchor point. These dockings suggest that the substrate and inhibitor adopt a mode of binding to *Pf*GCα comparable to that of 2′-deoxy-3′-AMP in MamAC, and the active sites appear relatively well conserved (Fig. 7A). Structural overlays indicate that the nucleobase occupies a highly similar position in the active sites (Fig. 7B). A few differences between the amino acids make it possible to accommodate a guanine base in *Pf*GCα instead of an adenine base in MamAC. Thus, while K938 in MamAC interacts with the N1 atom of adenine (as hydrogen bond acceptor), E3058 in *Pf*GCα can establish hydrogen bonds with the N1 atom and the amine function at the C2 position of guanine (as hydrogen bond donors) (Fig. 7A). MamAC D1018 interacts with the exocyclic amine of adenine, whereas the corresponding A3257 residue in *Pf*GCα allows no interaction with the oxygen in position C6 of guanine. Instead, this oxygen interacts with the main-chain amine of the adjacent residue (L3258). Baker (*46*) already highlighted that specificity for the nucleobase was due to the substitution of K578 and S684 in *Pf*AC to E3058 and A3257 in *Pf*GCα, respectively. We find that a third substitution seems necessary for guanine binding, as I940 of MamAC would clash with the exocyclic amine function of guanine (Fig. 7A). The corresponding residue is an isoleucine or a phenylalanine in other adenylyl cyclases (I580 in *Pf*ACα). In *Pf*GCα, the side chain of this residue (V3060) is shorter, allowing the exocyclic amine of guanine to be accommodated. Last, the two aspartates enabling interaction with phosphate groups (D396 and Asp440 in MamAC) are conserved in *Pf*GCα (D3975 and D4019). Interactions between *Pf*GCα and UA2239 are therefore predicted as plausible and show that UA2239 can replace GTP in the active site.

### Mechanisms of resistance to UA2239

**Fig. 8.**
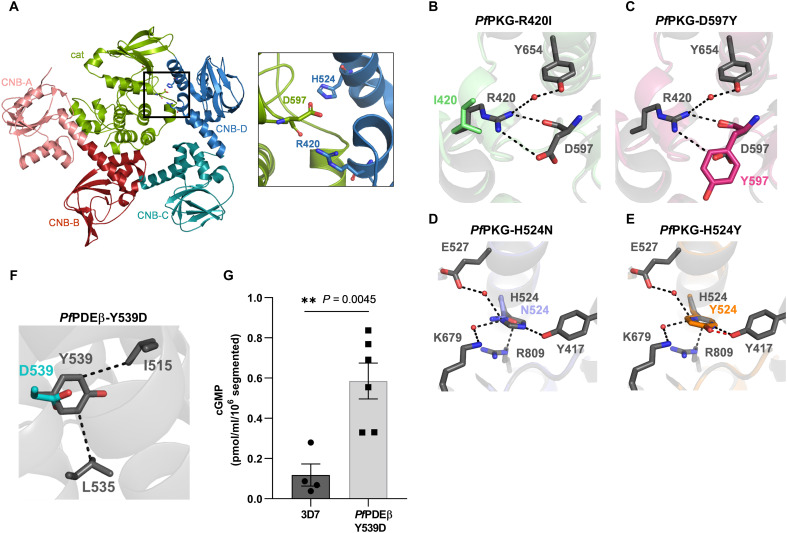
Mechanism of resistance to UA2239. (**A**) Localization of the mutated amino acids on the 3D structure of *Pf*PKG. Left: X-ray 3D structure of the full-length *Pf*PKG (PDB: 5DYK) (*39*) showing the mutated residues conferring resistance to UA2239 (black square) at the interface between the catalytic domain (green) and the CNB-D domain (blue). Right: close-up view of the interface showing the three mutated amino acids in the resistant strains. The colors of the domains are the same as in the schematic representation of the *Pf*PKG in Fig. 4A. (**B** to **E**) Close-up views of the superimposition of WT *Pf*PKG (gray) and the mutated *Pf*PKG (colored). [(B) and (C)] Interactions between R420 and D597 are lost in *Pf*PKG-R420I (green) and in *Pf*PKG-D597Y (pink). (D) Stacking between H524 and R809 is lost in *Pf*PKG-H524N (purple). (E) Hydrogen bounds between H524 and two water molecules are lost in *Pf*PKG-H524Y (orange). In addition, this mutation leads to a clash with Y417 shown as red dashed lines. (**F**) Close-up view of the superimposition of WT *Pf*PDEβ (gray) and the mutated *Pf*PDEβ-Y539D (cyan). Interactions between are Y539 and two hydrophobic residues, I515 and L535, are lost in the *Pf*PDEβ-Y539D mutant. (**G**) Intracellular cGMP levels in 3D7 and *Pf*PDEβ-Y539D GER-parasites. Experiments were conducted as for Fig. 6F. (*n* = 4 and 6 independent experiments for 3D7 and *Pf*PDEβ-Y539D, respectively, means ± SEM). Two-tailed Student’s *t* test was used to calculate *P* value.

Taking advantage of the available *Pf*PKG 3D structure (*39*, *47*), we analyzed the impact of the mutations R420I, H514N/Y, and D597Y that all render the parasite resistant to UA2239. The three amino acids are all located at the interface between the CNB-D regulatory domain and the catalytic domain (Fig. 8A). We generated structural models of each mutated *Pf*PKG using AlphaFold2 (*45*). The mutations did not induce significant conformational changes in the *Pf*PKG 3D structure (fig. S9A). However, they appear to be deleterious to interactions between CNB-D and the catalytic domain (Fig. 8, B to E). R420 is involved in water-mediated contacts with Y654 and in salt bridges with D597 of the catalytic domain (Fig. 8, B and C). Interactions between R420 and D597 are lost in R420I and D597Y mutants. The H524N mutation disrupts the stacking interaction between H524 and R809 (Fig. 8D). Interactions between H524 and two water molecules are lost in the H524Y mutant (Fig. 8E). In addition, this latter mutation leads to a steric clash with Y417 (Fig. 8E). Mutations of R420, H524, or D597 disrupt key interactions and are expected to destabilize the interface between the CNB-D domain and the catalytic domain of *Pf*PKG.

The Y539D mutation in *Pf*PDEβ was also analyzed using the AlphaFold model of the protein (fig. S10). Y539 is located within the transmembrane α helix bundle and is involved in hydrophobic interactions with I515 and L535 (Fig. 8F). Substituting tyrosine by aspartic acid at position 539 disrupts these hydrophobic interactions. To better understand this mechanism of resistance, we quantified the cGMP level in the PDEβ-Y539D GER–resistant strains. cGMP level increased in the mutated strain compared to 3D7 (Fig. 8G), indicating that the Y539D mutation impairs the cGMP hydrolysis activity of PDEβ.

## DISCUSSION

UA2239 belongs to the chemical family of ANPs. The advantages of these molecules lie in their chemical properties that make them very good candidates for the development of antimalarial agents. They are metabolically stable nucleotide analogs due to the presence of a phosphonate group, they can be synthesized in few steps from readily available and inexpensive chemicals, and they can be administered orally (*48*, *49*). Unexpectedly, the mode of action of UA2239 is totally different from those previously described for ANPs. When used in antiviral and anticancer therapies (*10*, *34*, *50*), the mechanism of action is linked to the inhibition of viral or cellular polymerases. In *Plasmodium*, already reported ANPs have been shown to inhibit the activity of recombinant enzymes of the purine salvage pathway (*31*–*33*). On the contrary, extensive phenotypic characterization carried out in this study demonstrated that UA2239 targets the egress of merozoites from the iRBC and also inhibits gametocyte egress and male gametogenesis. Egress is a finely tuned process controlled by *Pf*PKG, which is activated by cGMP. The intracellular cGMP level is regulated by its synthesis through *Pf*GCα and its degradation by *Pf*PDEs. The observed decrease in cGMP in the presence of UA2239 provides evidence that *Pf*GCα is likely to be the primary target. Thus, UA2239 does not target DNA polymerase as observed for ANPs in other organisms (*10*) but acts on a different enzyme. Structural similarities between prokaryotic DNA polymerase I and nucleotide cyclases have previously been suggested, which could explain the unexpected mechanism of action of UA2239 (*51*, *52*). Future work on the 3D structural determination of *Pf*GCα in complex with the compound may provide deeper insights into the impact of these shared structural features. *Pf*GCα has been shown to be the only enzyme producing cGMP in asexual blood stages (*17*). This enzyme has also been shown to be essential for parasite growth, and the effect of UA2239 on merozoite egress was similar to the one observed when *Pf*GCα was disrupted (*17*). UA2239 is a GMP analog, and our docking model shows that the compound can be accommodated and stabilized in the active site of the GC domain, supporting the idea that *Pf*GCα is the target of UA2239. Important amino acid differences between the *P. falciparum* and the mammalian enzymes may enhance the specificity of the compound for the parasite enzyme. In the catalytic sites, L3065 and A3257 of *Pf*GCα are not conserved in mammalian GCα but are both cysteine residues (*53*).

Despite this body of evidences, it cannot be ruled out that the drug may act on other regulators upstream of GCα activity. For example, the role of the adjacent P4-ATPase domain (*17*) or CDC50s (*54*) in regulating GC activity has not yet been fully elucidated. Interacting partners of *Pf*GCα, such as the unique GC organizer and the signaling linking factor, have been identified; both play important roles in up-regulating GC activity just before egress in the asexual blood stage (*55*). In addition, GCα has been identified as a substrate of the protein phosphatase PP1 (*56*) and is most likely phospho-regulated (*57*). Clearly, further studies are needed to decipher, at the molecular level, the mechanism of action of UA2239 on its target.

Resistance is not conferred by mutations in *Pf*GCα itself but in downstream effectors of the egress pathway. The effect of UA2239 on reducing cGMP levels can be circumvented by two resistance mechanisms. Mutations were found in the other two key enzymes of the cGMP signaling pathway, *Pf*PKG and *Pf*PDEβ. Mutations in *Pf*PKG and *Pf*PDEβ were validated as being responsible for the resistance mechanism. However, the mutated lines have an important fitness cost and are rapidly outcompeted by the WT strain in culture. While extrapolation from in vitro conditions to the field should be done with caution, it might be reasonable to assume that these mutations will not easily appear in the field. The mutations in *Pfpkg* and in *Pfpde*β conferring resistance to UA2239 were not found in the 16,203 *P. falciparum* field isolates from 33 countries available in the *Pf*7 dataset using the *Pf*-HaploAtlas app (*58*). In addition, the minimum inoculum for resistance (MIR) for UA2239 was found to be of 3.4 × 10^8^ parasites (see Materials and Methods section). These findings support the hypothesis that it can be difficult to generate resistance in the field.

Although UA2239 does not inhibit *Pf*PKG, resistance arises through mutations in this enzyme. *Pf*PKG is in an inactive conformation until the cytosolic level of cGMP increases. Cooperative binding of cGMP first to CNB-D and then to CNB-A and CNB-B changes the protein into its active conformation that in fine triggers the egress. This regulatory process is essential to ensure that merozoites egress neither too early nor too late in the cycle. Structural analysis of *Pf*PKG enabled visualization of the potential impact of the R420I, H524N/Y, and D597Y mutations. Modifications of these amino acids have already been studied. Franz *et al.* (*59*) showed that a D597N mutation resulted in threefold decrease in the activation constant of *Pf*PKG, indicating that mutating this amino acid causes destabilization of the inactive state of the enzyme. An H524A mutation also destabilizes the inactive *Pf*PKG apo-form (*39*). We suggest that the mutations identified here destabilize *Pf*PKG that therefore requires less cGMP than WT *Pf*PKG to switch from the inactive apo-form to the active holo-form. The four mutations in *Pf*PKG are thus expected to confer resistance through a compensatory process.

The only resistant clone that did not have a mutation in the *Pfpkg* gene was mutated in the *Pfpde*β gene. At this stage, we can only speculate that the mutation might induce a conformational change of *Pf*PDEβ and/or prevent binding of potential partners, leading in one way or another to the observed decrease in enzymatic activity, resulting in higher levels of cGMP, which are then sufficient to timely activate PKG in the presence of UA2239. Last, the GDV1 mutation emerged only in combination with the D597Y mutation in *Pf*PKG and the Y539D mutation in *Pf*PDEβ, both associated with the most severe fitness costs to the parasite. This GDV1 mutation may enable the parasite to conserve cellular resources by down-regulating gametocyte production, thereby enhancing its chances of survival under selective pressure.

Decades of studies have demonstrated the importance of the cGMP-PKG–dependent egress pathway in the malaria parasite and the potential for the involved proteins to be valuable therapeutic targets. Several PKG and PDE inhibitors have been extensively studied in recent years (*19*–*22*, *26*), but to our knowledge UA2239 is the first known GCα inhibitor. By disrupting the balance of cGMP in the parasite, which plays a crucial role at multiple stages of its life cycle, UA2239 interferes with the signaling pathway associated with this second messenger, leading to lethal effects. The pharmacological profile of UA2239 fits the criteria for promising antimalaria compounds as defined by Medicines for Malaria Venture. UA2239 is effective at low nanomolar concentration against sensitive and chemoresistant strains, is active in vivo in a mouse model, and targets at least two stages of the parasite life cycle. Its effect on reducing the level of cGMP and its irreversible action on merozoite egress and on gametogenesis have been established. Its ability to enter through NPP enables a specific effect on iRBCs. Its mechanism of action is new, although it has not yet been fully elucidated at the molecular level. UA2239 clearly provides a therapeutic advantage by irreversibly targeting parasite development and transmission to the mosquito, even when administered only during the first days of gametocytogenesis. This study revealed the antimalarial potential of UA2239 and its novel mechanism of action. It lays the foundation for developing an oral formulation with the aim of advancing UA2239 as a clinical candidate in the pipeline of antimalarial drugs.

## MATERIALS AND METHODS

### Parasite culture

*P. falciparum* asexual blood stage parasites were grown in O^+^ or A^+^ erythrocytes supplied by the local blood bank (Etablissement Français du Sang, Pyrénées Méditerranée, France). Cultures were at 5% hematocrit in complete medium, constituted of RPMI 1640 containing 25 mM Hepes (Gibco Life Technologies), supplemented with 10% of human AB^+^ serum and gentamicin (25 μg/ml; Sigma-Aldrich). Cultures were maintained at 37°C under a controlled tri-gas atmosphere of 5% O_2_, 5% CO_2_, and 90% N_2_. All parasite cultures used the 3D7 strain as the WT strain.

### *Plasmodium* growth inhibition assays

Drug effects on *P. falciparum* growth were measured in microtiter plates according to a modified Desjardins test (*9*, *60*). The compounds were dissolved in RPMI 1640 or dimethyl sulfoxide (DMSO) and then further diluted in culture medium. Parasite growth was assessed by measuring the incorporation of [^3^H]-hypoxanthine into nucleic acids as previously described (*61*). Suspensions (200 μl) of *P. falciparum*–infected erythrocytes (1.5% final hematocrit and 0.6% parasitemia) were incubated with various concentrations of drug for 48 hours. Then, 30 μl of [^3^H]-hypoxanthine (0.5 μCi per well) were added for an additional 24-hour period. The reactions were stopped by freezing at −80°C. Cells were lysed by thawing, and the parasite macromolecules including nucleic acids were recovered by harvesting the lysate on glass-fiber filter plates (Unifilter 96 GF/C, PerkinElmer) using a FilterMate cell harvester (Packard Instruments). The radioactivity was counted on a Microbeta2 counter (Packard Instruments Revvity). Radioactivity background was obtained from incubation of noninfected erythrocytes under the same conditions, and the value obtained was subtracted. Parasitemia was evaluated and expressed as percentage of the control (without drug). Results were expressed as IC_50_, which is the drug concentration leading to 50% inhibition of parasite growth and were determined by nonlinear regression analysis of the dose-inhibition curve using GraphPad Prism 8.3.0 software. Data were normalized to the level of incorporation in the untreated and in the RBC (negative control). The experiments were performed at least three times, each at least in duplicates.

For stage-dependent susceptibility, drug was added at various concentrations to synchronized cultures at ring (0 to 3 hpi), trophozoite (18 to 21 hpi), or schizont (36 to 39 hpi) stages. After incubation for 6 hours, the cells were washed three times and resuspended in fresh complete medium without drug and incubated until 52 hpi. Then, [^3^H]-hypoxanthine (0.5 μCi per well) was added for 24 hours, and the reaction was stopped by freezing the plates at −80°C. Cell viability was assessed following the above procedure for the drug sensitivity assay.

For time course assays, 750 nM UA2239 was added to synchronized cultures at the trophozoite (24 hpi) or schizont (36 hpi) stages. After incubation for various periods (0.5, 1, 2, 3, 4, and 6 hours), the cells were washed twice and resuspended in fresh complete medium. [^3^H]-hypoxanthine was added at 52 hpi and incubated for an additional 24 hours. Cell viability was assessed as described above.

### Interaction between drugs—Isobolograms

The interaction between UA2239 and other drugs—furosemide, CQ, and DHA—was studied using the isobologram method (*62*). The antimalarial activity of UA2239 was determined in the presence of the second drug at several concentrations that were lower than its IC_50_. The IC_50_ of each drug alone was found to be 100 μM for furosemide, 11 nM for CQ, and 2 nM for DHA. In these experiments, compound concentrations were expressed as a fraction of the IC_50_ of the compounds alone. These fractions were called fractional 50% inhibitory concentrations (FIC_50_). The type of interaction was determined by calculating the maximal sum of FIC_50_ of the two compounds combined for antimalarial characterization (ΣFIC_50_ = FIC_50_ UA2239 + FIC_50_ drug B). Isobolograms were constructed from the FIC_50_s of UA2239 and drug B at the tested fixed concentration ratios. A straight line represents additivity (ΣFIC_50_ ≥ 1 and < 2), a concave line denotes a trend toward synergy (ΣFIC_50_ < 1) or high synergy (ΣFIC_50_ ≤ 0.5), and a convex curve represents antagonism (ΣFIC_50_ ≥ 2) (*62*, *63*). In all experiments, UA2239 was diluted in serial one-third dilutions while the second compound was added at several fixed concentrations.

### Follow-up of parasite morphology and DNA content

After tight synchronization by the Percoll-sorbitol method, 0 to 2 hpi 3D7 rings 3D7 were treated with 2 μM UA2239. At intervals of 6 hours for over a period of 72 hours, thin blood smears were prepared and aliquots of infected erythrocytes were fixed in 4% paraformaldehyde (PFA) for 4 hours at room temperature (RT) before storage at 4°C. Just before analysis, the cells were washed twice with phosphate-buffered saline (PBS) and stained with 3.3× SYBR Green I (Invitrogen) for 30 min. Cells were washed once and resuspended in PBS. Fluorescence was measured with a Becton Dickinson FACS Canto 1 cytometer, and 100,000 events were recorded per sample. Data were analyzed with Diva and FlowJo softwares. Cellular DNA content correlates with the intensity of fluorescence.

### Immunofluorescence assays

PV1-GFP (*35*) infected erythrocytes at late schizont stage were incubated with UA2239 (750 nM), C2 (1.5 μM), or E64 (10 μM) for 4 hours. Then, thin blood smears were realized made and fixed with 4% PFA in PBS for 30 min at RT or overnight at 4°C in a humidified chamber. After quenching for 4 min with 0.1 M glycine in PBS, the cells were permeabilized with 0.1% Triton X-100/PBS for 5 min and incubated for 30 min with 1.5% of bovine serum albumin (BSA)/PBS solution to block unspecific binding. Primary rabbit anti-GFP antibody (Invitrogen, A-6455) was used at 1:4000 dilution for 1 hour, and the cells washed three times with PBS before incubation for 1 hour with Alexa Fluor 488 goat antirabbit secondary antibodies (1:10,000; Invitrogen). Nuclei were stained with Hoechst (1:10,000). Slides were mounted with vectashield medium for imaging. Images were taken on a Leica Thunder microscope at the Montpellier magnetic resonance imaging (MRI) facility. Images were processed by Fiji software for sectioning and contrast adjustment.

### Electron microscopy

Highly synchronized *P. falciparum* cultures (0 to 3 hours) were treated with 2 μM UA2239 at 6 or 36 hpi. At 44 to 46 hpi, iRBCs were purified on VarioMACS columns (Miltenyi Biotec). As controls, the same cultures were treated with C2 at 36 hpi or left untreated. Samples were fixed by adding 25% glutaraldehyde (electron microscopy grade) directly into the culture medium to obtain a final concentration of 2.5%. After 10 min at RT, the cells were centrifuged and the pellet resuspended in 20 pellet volumes of 0.1 M cacodylate buffer containing 2.5% glutaraldehyde and 5 mM CaCl_2_. The suspension was left 2 hours at RT and then kept at 4°C in the fixative until further processing. All the following incubation steps were performed in suspension, followed by centrifugation. Cells were washed with cacodylate buffer and postfixed with 1% OsO_4_ and 1.5% potassium ferricyanide in cacodylate buffer. After washing with distilled water, samples were incubated overnight in 2% uranyl acetate in water and dehydrated in graded series of acetonitrile. Impregnation in Epon 812 was performed in suspension in Epon:acetonitrile (50:50) and two times 1 hour in 100% Epon. After the last step, cells were pelleted in fresh Epon and polymerized for 48 hours at 60°C. All incubation and washing steps were performed using a microwave processor PELCO Biowave Pro+ (TED PELLA) except for the overnight incubation. One hundred–nanometer sections were made with an ultramicrotome Leica UCT collected on silicon wafers, contrasted with uranyl acetate and lead citrate and observed on a Zeiss Gemini 360 scanning electron microscope on the MRI EM4Bio platform under high vacuum at 1.5 kV. Final images were acquired using the Sense BSD detector (Zeiss) at a working distance between 3.5 and 4 mm. Independent random tiles were acquired with a pixel size of 5 nm and RBCs infected with segmented schizonts were counted. Representative pictures were acquired with a pixel size of 1 nm for illustration.

### Assays on *P. falciparum* gametocytogenesis and gametogenesis

*P. falciparum* parasites of the NF54 strain or the B10 clone (*64*) were cultivated under standard conditions using RPMI 1640 medium supplemented with 10% heat-inactivated human serum and human erythrocytes at a 5% hematocrit. Parasites were kept at 37°C in a tri-gas atmosphere. Synchronous production of gametocytes was achieved by treating synchronized cultures at the ring stage (10 to 1 % parasitemia, day 0) with 50 mM *N*-acetyl glucosamine (NAG) to eliminate asexual parasites. NAG was maintained for 5 days until no asexual parasites were detected in the culture. UA2239 (750 nM) was added to the medium at day 1 post–NAG addition, either for 24 hours (stage I) or for 10 days. To determine the effect on stage V, UA2239 was added at day 10 post–NAG addition for 24 hours. Giemsa-stained smears were scored daily for gametocyte density and distribution of gametocyte stages (stages I to V). At day 10 post–NAG, stage V gametocytes were subjected to gametogenesis assay (*8*). In brief, 200-μl samples from each condition were stained for 15 min at 37°C with wheat germ agglutinin (WGA, 5 μg/ml)–Alexa Fluor 647 and DNA intercalating dye Hoechst 33342 (0.5 μg/ml). Samples were then pelleted at 1000*g* for 1 min and then mixed in 50 μl of human serum and subjected to a temperature drop (from 37° to 22°C at RT) for 20 min during which gametogenesis took place. Samples were fixed either before or 20 min after activation of gametogenesis with 1% PFA for 30 min at RT. After one wash in PBS, stained cells were mounted in a microscope slide under a sealed coverslip. Samples were observed at 100× magnification using a Leica DMi8. At least 100 gametocytes were analyzed for each sample in four independent experiments. Percent rounding and gamete egress were determined by calculating the percentage of round gametes and of WGA-negative gametes (due to loss of host erythrocyte) in the total gametocyte population, respectively.

### Assays on gametogenesis after treatment of *P. berghei*–infected mice

This study is compliant to the national and European regulations and to French laws (EU directive no. 86/609 modified by the directive 2010/63 regarding the Protection of Animals used for Experimental and Other Scientific Purposes). The animal studies were performed at the “Centre d’Elevage et de Conditionnement Experimental des Modèles Animaux,” Montpellier, under permission no. C3417234 (University of Montpellier) after approval by the animal experimenting commission (project no. APAFIS #39640-2022120118201950 v4). Gametogenesis was evaluated by counting the number of exflagellation centers in blood of female Naval Medical Research Institute (NMRI) mice infected with *P. berghei* ANKA at a parasitemia of 15 to 30%. For each mouse, a drop of blood was taken from the tip of the tail and placed between a slide and a coverslip, before (control) and 30 min after intraperitoneal treatment with UA2239. After 10 min at 20°C, exflagellation centers were counted under the microscope and the number of exflagellation centers per 10,000 cells was determined. The counting was done twice for the control before UA2239 treatment. Activity of UA2239 is expressed as the number of exflagellation centers per 10,000 cells as a percentage of control.

### Generation of UA2239 resistant strains and analysis of WGS

We used two different protocols to obtain UA2239-resistant *P. falciparum* blood-stage parasites. Drug pressure was applied intermittently by drug-on/drug-off cycles either using the same high concentration (10 × IC_50_) or gradually increasing the concentration of UA2239 (from 3 × to 10 × IC_50_). Parasites were cultured in the presence of UA2239 until parasites were no longer detectable on thin blood smears. The cells were then washed and cultured in the absence of drug until parasitemia reached 1% and another cycle of drug pressure began. After each cycle, parasite sensitivity to UA2239 was determined. Parasites were cultured independently and in triplicates for 7 months. From all six independent and resistant populations, several clones were isolated. The 3D7 strain was cultured in parallel during the 7 months and was also included in the WGS, but as a population. Sequencing was done by MGX-Montpellier GenomiX in two independent runs on Illumina MiniSeq platform. The 150–base pair (bp) paired-end sequencing reads from both runs were merged for each sample to increase the sequencing depth. Sequence adapters were trimmed with Trimmomatic (version 0.39), and filtered reads were mapped on the 3D7 reference genome (version 46) with bwa 0.17 (*65*, *66*).

SNPs and micro-INDELs were found with GATK (version 4.2.0.0) HaplotypeCaller, CombineGVCFs and GenotypeGVCFs (*67*). SNPs and micro-INDELs were discarded from a sample if they were covered by less than five reads. As each sequenced sample originates from a clonal population, only homozygous variant calls (at least 90% of the sample reads supporting one allele) were considered. SNPs and micro-INDELs with a mutated allele present in a drug-resistant sample and absent from the drug-sensitive sample were extracted using varif [available at Zenodo (https://doi.org/10.5281/zenodo.17541131) or GitHub (https://github.com/marcguery/varif, version 0.5.4)]. Macro-INDELs, duplications, inversions, and translocations were found and filtered with DELLY (version 0.8.7) using the options “-p -f somatic -a 0.5 -r 0.5 -v 10 -c 0.01” (*68*). Haplotypes of selected proteins of interest were screened with *Pf*-HaploAtlas in the 16203 *P. falciparum* samples available in the Pf7 dataset (*58*, *69*).

### Generation of transgenic 3D7 lines with single amino acid changes in *Pf*PKG by CRISPR-Cas9

The *Pf*3D7 *pkg* locus consists of five exons spanning 3561 bp encoding a protein of 853 amino acids. The mutations R420I, H524N/Y, and D597Y identified in the UA2239-resistant strains by the WGS and a previously identified T618Q mutation rendering parasites resistant to the PKG inhibitor C2 are all located in exon 5 (fig. S3). A sequence of 675 bp covering all these positions (position 2212 to 2886 of the *pkg* locus) was recodonized (termed ePKG). Six synthetic DNA fragments were ordered, five of which encode a single of the above listed mutations and one without mutation. Homology regions HR1 (510 bp) and HR2 (658 bp) for double homologous recombination were both cloned together into a KpnI/SacI-digested pBluescript plasmid. This created an EcoRV site between HR1 and HR2 regions, which we then used to insert the recodonized sequences. Plasmid cloning were done by In-Fusion and verified by Sanger sequencing (Eurofins Genomics). Three different guide RNA sequences (g12, g13, and g15) were identified in the 675-bp sequence using ChopChop (*70*). Complementary primer pairs were designed, annealed, and ligated into BbsI-digested plasmid pDC2-cas9-sgRNA-hDHFR/yfcu (*71*). The pDC2 plasmid and the pBluescript construct (75 μg each) were transfected together using ring stage cultures of 3D7 at about 5% parasitemia using a BioRad Gene Pulser (950 V, 310 μF, 200 ohm) (*72*). Selection with 2.5 nM WR22910 (gifted by Jacobus Pharmaceuticals) was initiated 5 hours after transfection and was maintained during daily media changes for 1 week. Genome-edited parasites were obtained with one of the three guide RNAs used. Parasite populations were cloned by limiting dilution.

### Generation of transgenic 3D7 lines with single amino acid changes in *Pf*PDEβ by CRISPR-Cas9

A mutant parasite line with a single amino acid change in the *Pf*PDEβ was generated using the same strategy as described above for the PKG mutations. The genomic locus of *pde*β is 4677-bp long, consists of nine exons, and encodes for a protein of 1139 amino acids (PF3D7_1321500). The mutation conferring resistance to UA2239 is located in exon 1. A sequence of 150 bp around the Y539D mutation (position 1513 to 1662) was recodonized and synthesized either in its mutated or WT version (named *Pf*ePDEβ) (fig. S4). HR1 (position 958 to 1512) and HR2 (position 1663 to 2165), were amplified and cloned together into a KpnI/SacI-digested pBluescript plasmid. This created an HincII restriction site between the HR1 and HR2 regions, which we then used to insert the recodonized sequences. Plasmid cloning were done by In-Fusion and verified by Sanger sequencing (Eurofins Genomics). One guide RNA sequence (g19) was identified in the 150-bp sequence using ChopChop. Complementary primer pairs were designed, annealed, and ligated into BbsI-digested plasmid pDC2-cas9-sgRNA-hDHFR-yfcu and transfected into ring stages of 3D7 as described above.

### Growth competition assay

To quantify the growth of GER-parasites in competition with 3D7 WT parasites, each GER strain was mixed with the 3D7 at equal parasitemia in 5-ml dishes and maintained in culture for a total of 40 days. Aliquots were collected regularly, and DNA was extracted using the DNA blood extraction kit (Qiagen). qPCR was performed using the LightCycler 480 Sybr Green I system (Roche) and the LightCycler 480 Software, and version 1.5 was used for “absolute fit point” data analysis. The strains could be distinguished through the presence of recodonized *epkg* sequences in all GER-parasite lines. Forward primer AGGTGCCACGGCCGATTATGC and reverse primer GCTACTGTATGATGAACCGC were specific for all GER-parasites. Forward primer CAAGGACCTATGTTAGCACATTTG and reverse primer CTAATTTAACTGTTCCGAAAGTACCTCTT were specific for 3D7 *Pfpkg* sequence. To validate that the presence of recodonized sequence in the *pkg* gene did not in itself induce a fitness cost, we also performed a competition assay between 3D7 and the ePKG-WT GER-parasite clone. No effect of recodonisation on the parasite fitness was observed (fig. S11).

### Ring-stage survival assay

After tight synchronization by the Percoll-sorbitol method, 0 to 3 hpi rings were diluted to 0.5 to 1% parasitemia and 2% hematocrit as previously described (*73*). Parasites were exposed to 0.01% of DMSO (control) or to 700 nM DHA for 6 hours at 37°C in a tri-gas atmosphere in duplicates for each condition and strain. Then, the cultures were washed and cultivated for an additional 66 hours. Culture medium was changed daily. The parasitemia was determined on blood smears by counting at least 10,000 cells. Survival rates were calculated as the ratio of parasites in treated and untreated cultures. A strain is considered resistant to artemisinin if the survival rate is higher than 1%. As positive control for resistance, the *P. falciparum* NF54 strain with the C580Y substitution in Kelch 13 (*74*) was used (gifted by J.-J. Lopez-Rubio, LPHI). *P. falciparum* artemisinin-sensitive 3D7 and NF54 strains were used as negative controls.

### Enzymatic assay of recombinant *Pf*PKG

The full-length *Pf*PKG was expressed and purified by the Protein Biochemistry Platform of the faculty of medicine, Geneva University. Baculoviruses expressing the *Pf*PKG were generated from a modified pFastBac vector–like vector for insect cell expression. It contains an N-terminal “TwinSTREP-His-TEV” tag. Protein samples were first purified by affinity using STREP-tactin resin. A second step of purification was done after TEV cleavage by size exclusion chromatography using a Superdex 200 Gel Filtration column equilibrated in 20 mM tris (pH 7.4), 150 mM NaCl, and 3 mM dithiothreitol (DTT). *Pf*PKG inhibition assays were performed on the basis of previously described methods (*22*), using adenosine 5′-diphosphate (ADP) formation as a measure of kinase activity. The reaction buffer for all measurements consists of 25 mM Hepes (pH 7.4), 0.01% (w/v) BSA, 0.01% (v/v) Triton X-100, 20 mM MgCl_2_, 2 mM DTT, 10 μM cGMP, 10 μM adenosine 5′-triphosphate (ATP), 20 μM peptide substrate GRTGRRNSI (GeneCust), and 50 nM purified *Pf*PKG protein. UA2239 was added at 1, 10, or 100 μM and C2 at 0.1 or 1 μM. Reactions were initiated by the addition of the enzyme and incubated for 30 min at RT. ADP formation was measured using the ADP-Glo Kinase Kit (Promega) according to the manufacturer’s instructions. Briefly, 25 μl of ADP-Glo Reagent was added to 25 μl of kinase reaction in white 96-well plates (PerkinElmer) and incubated for 40 min at RT to deplete the remaining ATP. Then, 50 μl of kinase detection reagent was added, and the reaction was incubated for a further 30 min at RT. Luminescent signal was measured using the Sunrise Microplate Reader (Tecan).

### Expression and purification of *Pf*PKG cGMP-binding domains and ITC experiments

The CNB-A (21 to 158) and CNB-D (401 to 542) domains were cloned into the pETM11 vector. Protein constructs were expressed in *Escherichia coli* BL21(DE3) grown in LB medium supplemented with kanamycin (50 μg/ml) at 37°C. Bacterial growth was monitored by measuring optical density at 600 nm until it reached 0.6 to 0.8. The culture was then cooled to 18°C for 1 hour before induction with 0.5 mM isopropyl-β-d-thiogalactopyranoside. Protein expression was carried out overnight at 18°C with continuous shaking at 200 rpm. Cells were harvested the following day by centrifugation at 7000*g* for 15 min and resuspended in lysis buffer [25 mM Hepes (pH 8.0), 300 mM NaCl, and 10 mM imidazole for CNB-A and 25 mM Hepes (pH 7.5), 500 mM NaCl, 5% (w/w) glycerol, and 10 mM imidazole for CNB-D] supplemented with a protease inhibitor cocktail (cOmplete, Roche). Cell lysis was achieved by sonication in three cycles of 5 min (50% duty cycle and 65% amplitude), with 5-min cooling intervals on ice between cycles. The total lysates were centrifuged at 16,000*g* for 1 hour at 4°C. The supernatant containing the soluble CNB-A and CNB-D were subjected to a two-step purification process. Affinity purification was performed on a 1-ml HisTrap HP column (Cytiva) using an ÄKTA Pure high-performance liquid chromatography (HPLC) system operated at a flow rate of 1 ml/min. The recombinant proteins were eluted in their own lysis buffer supplemented with 250 mM imidazole. Eluates were further purified by size exclusion chromatography using a HiLoad 16/60 Superdex 75 pg column (GE Healthcare) on the same HPLC system. Elution buffers were 25 mM Hepes (pH 8.0) and 300 mM NaCl for CNB-A, and 25 mM Hepes (pH 7.5), 500 mM NaCl, and 5% (w/w) glycerol for CNB-D. Fractions with the highest concentration and purity of recombinant proteins were pooled and concentrated using a centrifugal filter unit (Amicon, Merck) with a 10-kDa molecular weight cutoff, and subsequently used for ITC experiments.

The dissociation constants and stoichiometries of cGMP or UA2239 binding to CNB-A or CNB-D were determined using a PEAQ-ITC microcalorimeter (Malvern Panalytical Ltd). Protein concentrations were 50 and 10 μM for CNB-A and CNB-D, respectively. Each experiment was performed by loading 200 μl of purified protein, into the sample cell. CNB-A was titrated with 500 μM cGMP or UA2239, whereas CNB-D was titrated with 100 μM cGMP or UA2239. The injection syringe was filled with 40 μl of cGMP or UA2239. For CNB-D, 34 injections of 1.5 μl of cGMP or 18 injections of 2 μl of UA2239 were delivered at 150-s intervals. For CNB-A, 25 injections of 1 μl of cGMP or 15 injections of 2 μl of UA2239 were delivered at 150-s intervals. All ITC measurements were conducted at 25°C with constant stirring at 500 rpm. The raw data were fitted by using the MicroCal PEAQ-ITC analysis software (v. 1.41, Malvern Panalytical Ltd) using the single-site binding model to estimate the binding parameters.

### Measurement of intracellular cGMP and cAMP levels

cGMP and cAMP levels were measured using Direct cGMP or cAMP enzyme-linked immunosorbent assay kit (Enzo Life Sciences). Mature schizonts at 36 hpi were purified on VarioMACS columns (Miltenyi Biotech). Then, approximately 1.5 × 10^8^ parasites were incubated in presence of 1.5 μM C2 (to prevent schizont rupture), without or with various UA2239 concentrations. At 42 hpi, parasites were collected by centrifugation (1 min at 600*g*) and resuspended in 100 μl of 0.1 M HCl. After 3 min of incubation at RT, samples were centrifuged for 5 min at 9000*g*. Supernatants were collected, diluted by addition of 200 μl of 0.1 M HCl, and frozen at −80°C. cGMP or cAMP levels were measured using the acetylated protocol according to the manufacturer’s instructions. For the experiments with the PDE inhibitor BIPPO, the compound was added to samples with C2 only or with C2 and 750 nM UA2239 at 42 hpi for 3 min, then collected by centrifugation (1 min at 600*g*), and processed as the other samples. Absorbance was detected at 405 nm on a Spark spectrophotometer (Tecan). The data were analyzed using GraphPad Prism 8.3.0.

### Treatment of parasite cultures with PET-cGMP

Synchronous late schizonts were treated with 750 nM UA2239 or 1.5 μM C2 from 38 hpi. At 44 hpi, 60 μM PET-cGMP was added or not to both cultures. Giemsa-stained thin blood smears were taken after 2 hours, and parasitemia of segmented schizonts and rings were determined (at least 5000 cells were counted).

### Generation of the 3D models and docking

The structure of the GC domain of *Pf*GCα (residues 2741 to 4226; fig. S8) was modeled using AlphaFold2 implemented on the CBS local server. The cyclase domain of this model was aligned with that of MamAC (PDB 1CS4, chains A and B), as shown in fig. S8 (B to D). The RMSD between the cyclase domains was calculated from residues 3007 to 3314 and 3963 to 4151 of *Pf*GCα GC domain, excluding two insertions. Dockings of UA2239 and GTP into the GC domain of *Pf*GCα were performed with PLANTS 1.2 (*75*), using the coordinate of the C1′ atom of 2′-deoxy-3′-AMP in the 1CS4 structure and a shape constraint of −3. The binding site radius was fixed to 10 Å. The 10 best-ranked poses were similar, except for the positions of the phosphates and the ribose for GTP which fluctuates. The structures of the mutated *Pf*PKG (R420I, H524N, H524Y, and D597Y) and *Pf*PDEβ (Y539D) were modeled using AlphaFold2 implemented on the CBS local server.

### Determination of the MIR

UA2239 resistance selection was performed by culturing 3D7 parasites continuously under 3 × IC_90_ (600 nM) drug pressure at 3% hematocrit in petri dishes as previously described (*76*). Starting parasite inocula were 1.28 × 10^7^ (three dishes) or 1.28 × 10^8^ (five dishes). Drug-containing media were replaced every day for the first 5 days and then every 3 or 4 days. RBCs were replenished by replacing half of the culture once a week. Cultures were monitored by Giemsa staining and microscopy daily until the parasites were cleared and then twice per week to detect recrudescence. Selections were maintained for 60 days or until recrudescent parasites were observed. The MIR value is defined as the minimum number of parasites used to obtain resistance and calculated as follows: total number of parasites inoculated divided by the total number of positive cultures. This formula includes lower inocula where there were no positive wells and excludes higher inocula in cases where lower inocula already yielded resistance. In this experiment, we observed recrudescence in only two dishes of 1.28 × 10^8^ parasites; consequently, the MIR value was calculated as follows: $\mathrm{MIR}=\frac{\left(3\;\times \;1.28\;\times \;10^{7}\right)+\left(5\;\times \;1.28\;\times \;10^{8}\right)}{2}=3.392\;\times \;10^{8}$ parasites.

### Statistical analysis

All statistical tests were performed using GraphPad Prism version 8.3.0. A Kruskal-Wallis nonparametric test was performed for Fig. 3E. Two-tailed unpaired Student’s *t* tests were performed for Figs. 3F and 6, A, F, and G. Paired-ratio Student’s *t* tests were performed for Fig. 6H. For WGS, all the samples were sequenced in two independent runs, and both runs were merged for each sample to increase the average depth. For the effect of UA2239 on the rupture of the PVM by electron microscopy, 72 to 195 segmented schizonts were analyzed. For the effect of UA2239 on *P. falciparum* gametocytes development, at least 100 gametocytes were analyzed for each sample in three independent experiments.

## Supplementary Materials

### This PDF file includes:

Figs. S1 to S11 Table S1 References
