# Supplementary material for: An acyclic nucleoside phosphonate effectively blocks the egress of the malaria parasite by inhibiting the synthesis of cyclic GMP
Source: Sci Adv. 2025 Nov 21;11(47):eady2859. doi: 10.1126/sciadv.ady2859 (PMC12637298; doi:10.1126/sciadv.ady2859)
Supplement: Supplementary file 1 — Figs. S1 to S11 Table S1 References [file sciadv.ady2859_sm.pdf]

Science Advances
AAAS

Supplementary Materials for

An acyclic nucleoside phosphonate effectively blocks the egress of the malaria parasite by inhibiting the synthesis of cyclic GMP

Marie Ali *et al.*

Corresponding author: Sharon Wein, sharon.wein-gratraud@umontpellier.fr; Rachel Cerdan, rachel.cerdan@umontpellier.fr

Sci. Adv. **11**, eady2859 (2025)
DOI: 10.1126/sciadv.ady2859

This PDF file includes:

Figs. S1 to S11
Table S1
References

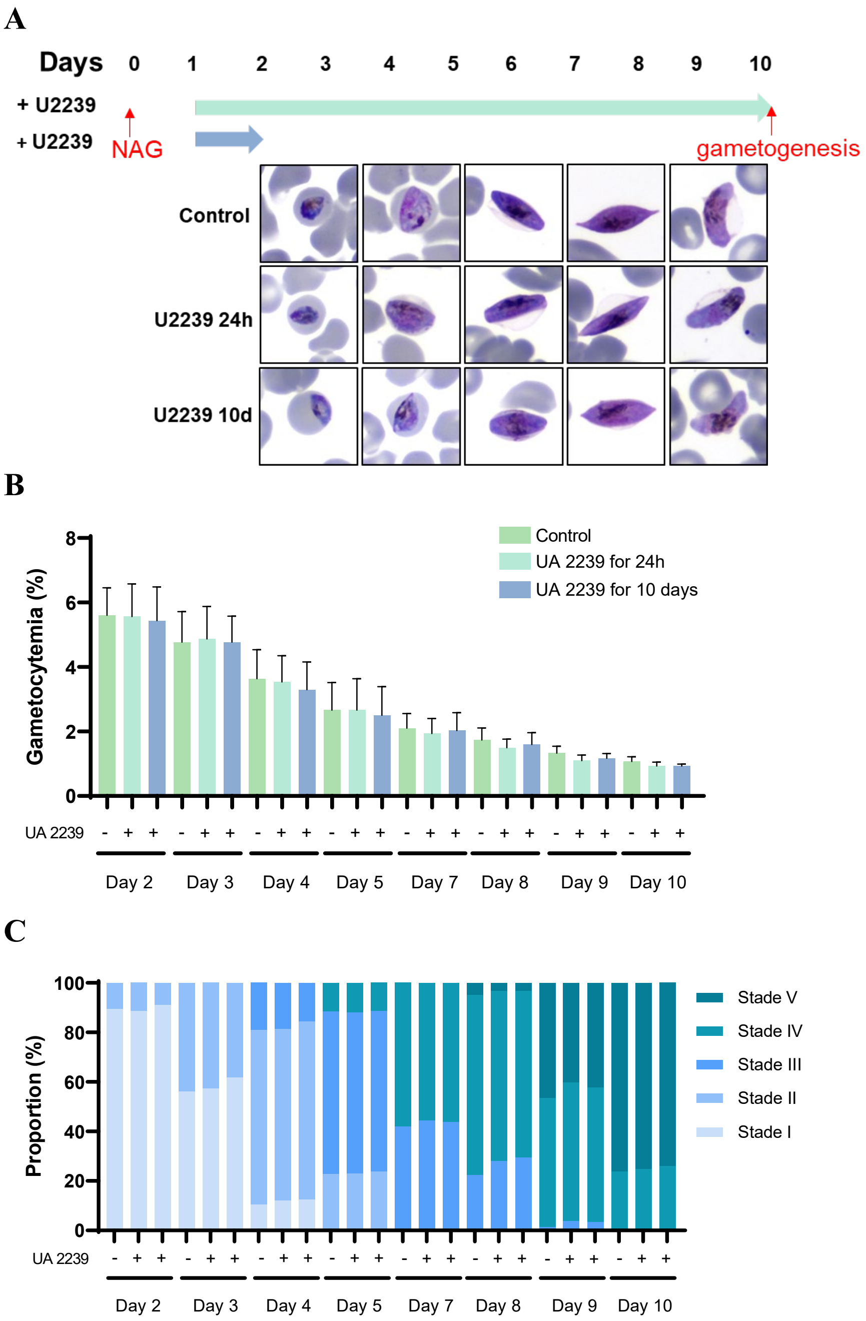

Fig. S1. Effect of UA2239 on gametocytemia. UA2239 was added or not on B10 cultures (NF54-derived clone) at day 1 post N-acetyl glucosamine (NAG) for 24 hours or for 10 days. Gametocytemia was followed during the 10 days for the 3 conditions. **(A)** Giemsa-stained smears of treated and untreated gametocytes. **(B)** Gametocytemia. **(C)** Proportions of the different stages of gametocytes up to 10 days of maturation (conditions in the same order as in B).

A

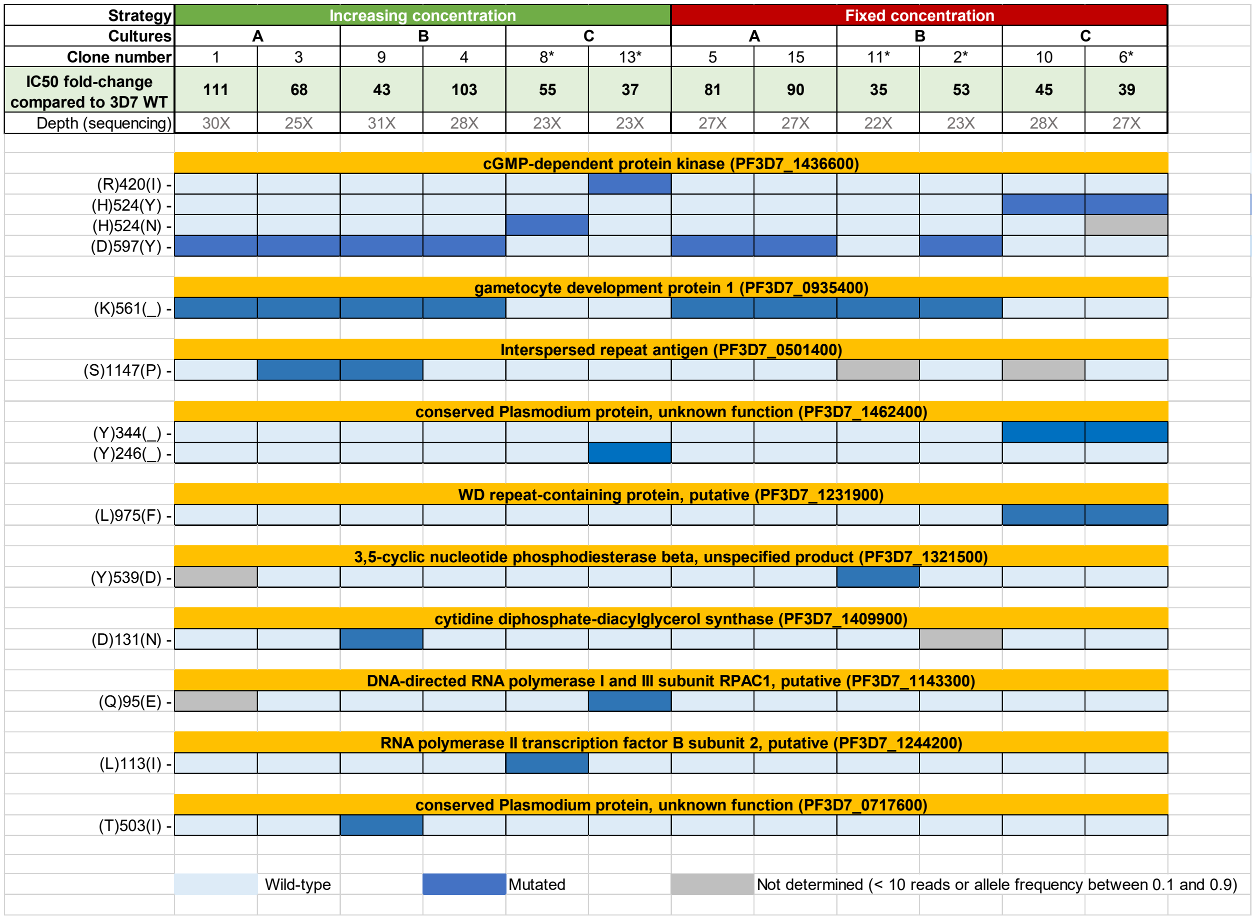
Strategy
Increasing concentration
Fixed concentration
Cultures
A
B
C
A
B
C
Clone number
1
3
9
4
8*
13*
5
15
11*
2*
10
6*
IC50 fold-change compared to 3D7 WT
111
68
43
103
55
37
81
90
35
53
45
39
Depth (sequencing)
30X
25X
31X
28X
23X
23X
27X
27X
22X
23X
28X
27X
cGMP-dependent protein kinase (PF3D7_1436600)
(R)420(I) -
(H)524(Y) -
(H)524(N) -
(D)597(Y) -
gametocyte development protein 1 (PF3D7_0935400)
(K)561(_) -
Interspersed repeat antigen (PF3D7_0501400)
(S)1147(P) -
conserved Plasmodium protein, unknown function (PF3D7_1462400)
(Y)344(_) -
(Y)246(_) -
WD repeat-containing protein, putative (PF3D7_1231900)
(L)975(F) -
3,5-cyclic nucleotide phosphodiesterase beta, unspecified product (PF3D7_1321500)
(Y)539(D) -
cytidine diphosphate-diacylglycerol synthase (PF3D7_1409900)
(D)131(N) -
DNA-directed RNA polymerase I and III subunit RPAC1, putative (PF3D7_1143300)
(Q)95(E) -
RNA polymerase II transcription factor B subunit 2, putative (PF3D7_1244200)
(L)113(I) -
conserved Plasmodium protein, unknown function (PF3D7_0717600)
(T)503(I) -
Wild-type
Mutated
Not determined (< 10 reads or allele frequency between 0.1 and 0.9)

B

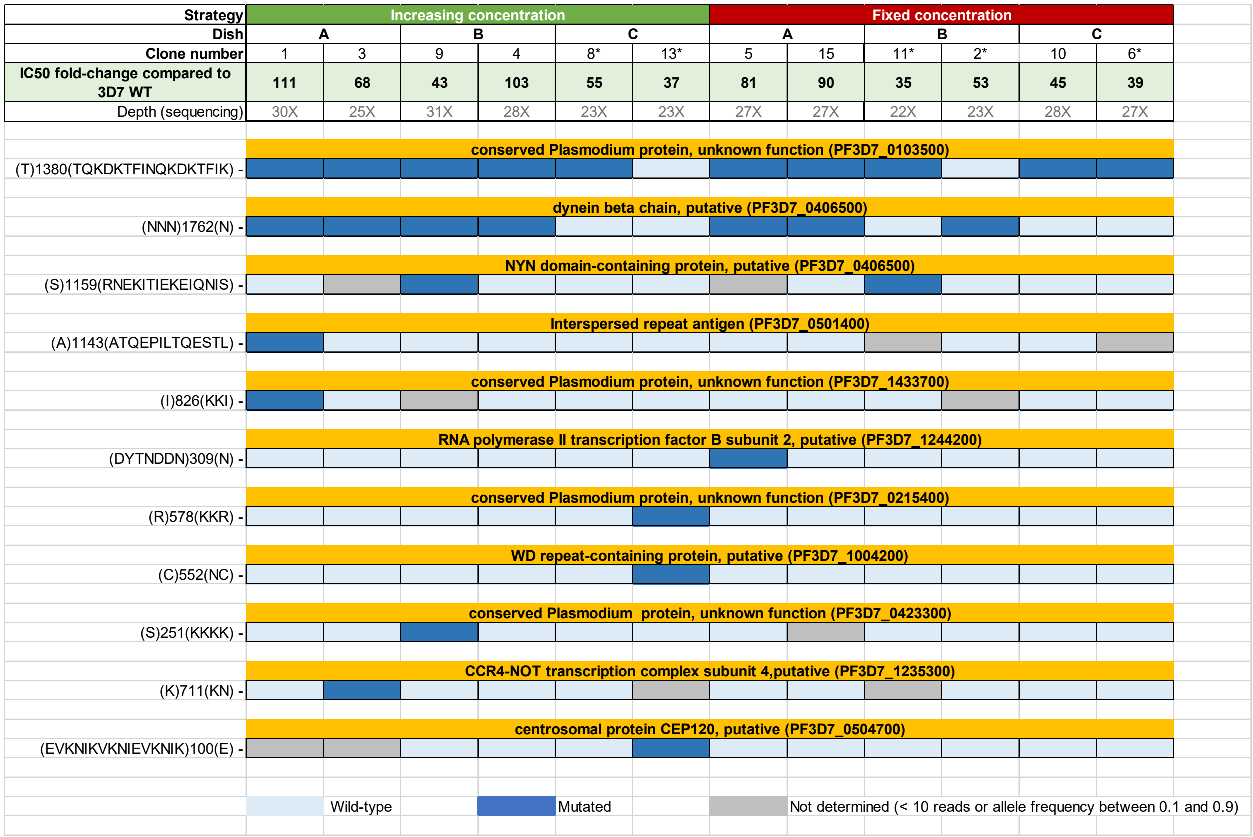

Fig. S2. Selection of clones and whole genome sequencing of resistant parasites.
Two clones per population of resistant parasites (R-parasites) were selected for whole-genome sequencing (12 clones in all), along with the 3D7 strain maintained in culture for the period required to obtain resistant parasites (sensitive parasites). Clone numbers are indicated, as well as the method used to obtain resistant parasites (exposure to an increasing or fixed concentration of drug), their IC_{50} fold-change (ratio of IC_{50} between resistant and WT parasites) and sequencing depth. Only variants (SNPs and micro INDELs) detected in one or more drug-resistant clones and absent in the drug-sensitive parasites were kept. **(A)** 14 single nucleotide polymorphisms (SNPs) leading to non-synonymous mutations. The four clones mutated in the *Pfpkg* gene and the clone mutated in the *Pfpdeβ* gene, chosen for further studies, are labelled with an asterisk. **(B)** 11 micro INDELs.

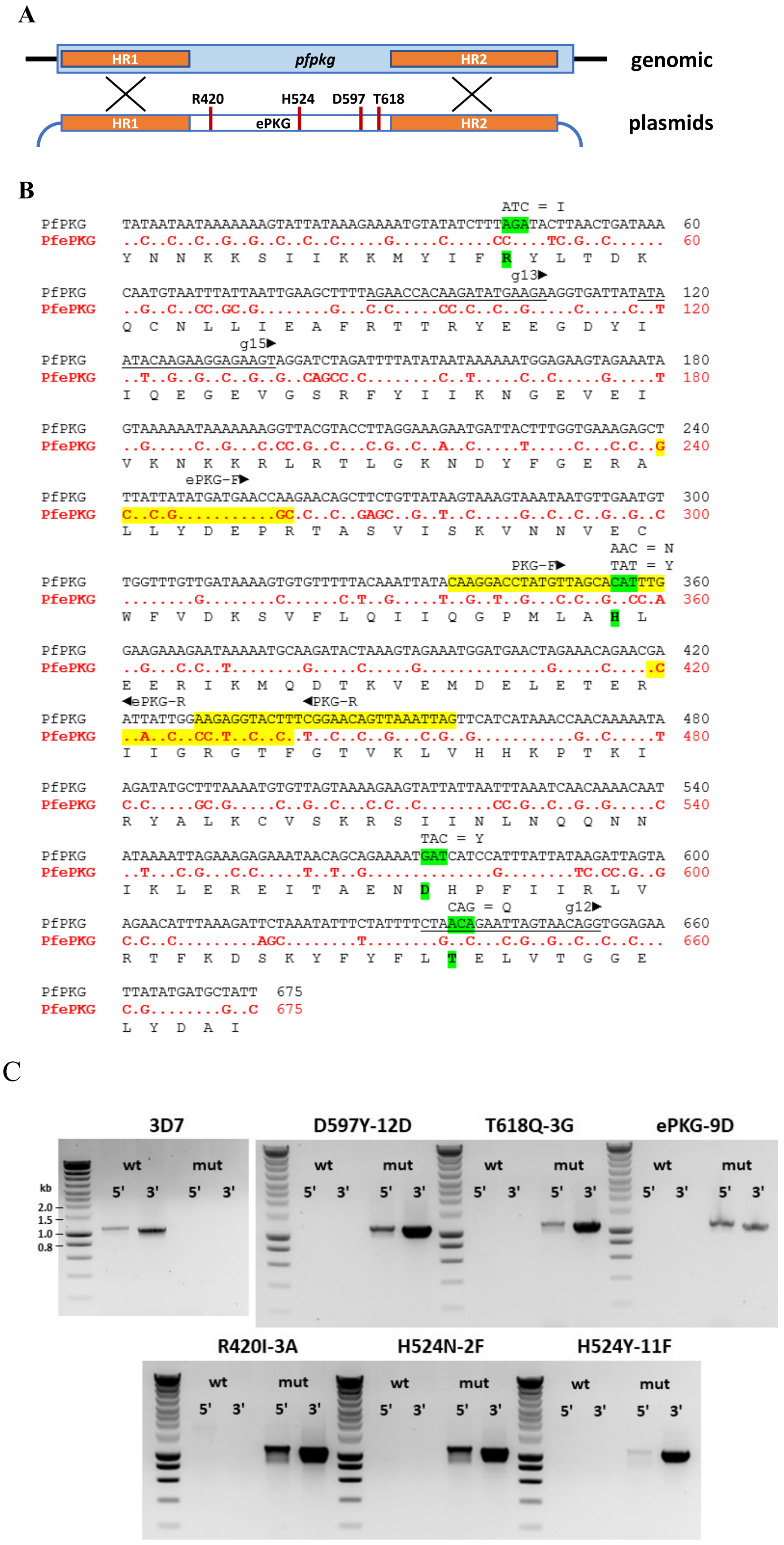
A
HR1
pfpkg
HR2
genomic
R420
H524
D597
T618
HR1
ePKG
HR2
plasmids
B
ATC = I
PfPKG TATAATAATAAAAAAAGTATTATAAAGAAAATGTATATCTTTAGATACTTAACTGATAAA 60
PfePKG ..C..C..C..G..G..C..C..C.....G.....C.....CC....TC.G..C...... 60
Y N N K K S I I K K M Y I F R Y L T D K
g13▶
PfPKG CAATGTAATTTATTAATTGAAGCTTTTAGAACCACAAGATATGAAGAAGGTGATTATATA 120
PfePKG ..G..C..CC.GC.G........G...C.C.....CC.C..C..G.....C.....C..T 120
Q C N L L I E A F R T T R Y E E G D Y I
g15▶
PfPKG ATACAAGAAGGAGAAGTAGGATCTAGATTTTATATAATAAAAAATGGAGAAGTAGAAATA 180
PfePKG ..T..G..G..C..G..G..CAGCC.C........C..T.....C..C.....G.....T 180
I Q E G E V G S R F Y I I K N G E V E I
PfPKG GTAAAAAATAAAAAAAGGTTACGTACCTTAGGAAAGAATGATTACTTTGGTGAAAGAGCT 240
PfePKG ..G.....C..G...C.CC.G..C...C.G..C..A..C.....T.....C...C.C..G 240
V K N K K R L R T L G K N D Y F G E R A
ePKG-F▶
PfPKG TTATTATATGATGAACCAAGAACAGCTTCTGTTATAAGTAAAGTAAATAATGTTGAATGT 300
PfePKG C..C.G..........GC.C..C..GAGC..G..T..C.....G..C..C..G..G..C 300
L L Y D E P R T A S V I S K V N N V E C
AAC = N
PKG-F▶ TAT = Y
PfPKG TGGTTTGTTGATAAAAGTGTGTTTTTACAAATTATACAAGGACCTATGTTAGCACATTTG 360
PfePKG ........G........C......C.T..G.....T..G..T..G...C.C..G..CC.A 360
W F V D K S V F L Q I I Q G P M L A H L
PfPKG GAAGAAAGAATAAAAATGCAAGATACTAAAGTAGAAATGGATGAACTAGAAACAGAACGA 420
PfePKG ..G...C.C..T........G.....C.....G..............G.....C.....C 420
E E R I K M Q D T K V E M D E L E T E R
◀ePKG-R ◀PKG-R
PfPKG ATTATTGGAAGAGGTACTTTCGGAACAGTTAAATTAGTTCATCATAAACCAACAAAAATA 480
PfePKG ..A..C..CC.T..C..C..T..C..C..G...C.G..G...........G..C.....T 480
I I G R G T F G T V K L V H H K P T K I
PfPKG AGATATGCTTTAAAATGTGTTAGTAAAAGAAGTATTATTAATTTAAATCAACAAAACAAT 540
PfePKG C.C.....GC.G.....C..G..C...C.C..C........CC.G..C..G..G.....C 540
R Y A L K C V S K R S I I N L N Q Q N N
TAC = Y
PfPKG ATAAAATTAGAAAGAGAAATAACAGCAGAAAATGATCATCCATTTATTATAAGATTAGTA 600
PfePKG ..T...C.G...C.C.....T..T..G...............G........TC.CC.G..G 600
I K L E R E I T A E N D H P F I I R L V
CAG = Q g12▶
PfPKG AGAACATTTAAAGATTCTAAATATTTCTATTTTCTAACAGAATTAGTAACAGGTGGAGAA 660
PfePKG C.C..C.........AGC........T.........G..C...C.G..G..C..C..C... 660
R T F K D S K Y F Y F L T E L V T G G E
PfPKG TTATATGATGCTATT 675
PfePKG C.G........G..C 675
L Y D A I
C
3D7
D597Y-12D
T618Q-3G
ePKG-9D
wt
mut
5'
3'
kb
2.0
1.5
1.0
0.8
R420I-3A
H524N-2F
H524Y-11F

Fig. S3. **Generation of transgenic parasites carrying mutations in *Pf*PKG**. (A) Schematic representation of the last exon (exon 5, blue box) of the *Pfpkg* locus (above) and of the transfection constructs (below). The homology regions (HR) for double homologous recombination and the position of the mutated residues are indicated. The recodonised sequence is named ePKG. (B) Alignment of the *Pfpkg* wild type sequence (top) and the ePKG in red (below). Dots indicate identical bases. The amino acid sequence is shown at the bottom. Mutated residues are highlighted in green and the modified sequence in the mutants shown above. The three guide RNA sequences (g12, g13, and g15) that led to the generation of the mutants are underlined. Primer sequences that were used in PCR to differentiate between wild type and transgenic parasites are indicated in yellow. (C) Genotyping of transgenic parasite clones carrying mutations in *Pf*PKG. Correct 5'- and 3' homologous recombination was detected by PCR on genomic DNA. For 5' integration primer Int-F in the sequence upstream of the HR1 region was used in combination with either the PKG-R (wild type) or ePKG-R (mutant, recodonised) primer. For 3' integration primer Int-R in the sequence downstream of HR2 was used in combination with either PKG-F (wt) or ePKG-F (mut). ePKG-9D corresponds to the transgenic clone carrying the recodonised sequence without any encoded amino acid change. SmartLadder (Eurogentec) was used as DNA molecular weight marker and relevant bands are indicated in kilobases (kb).

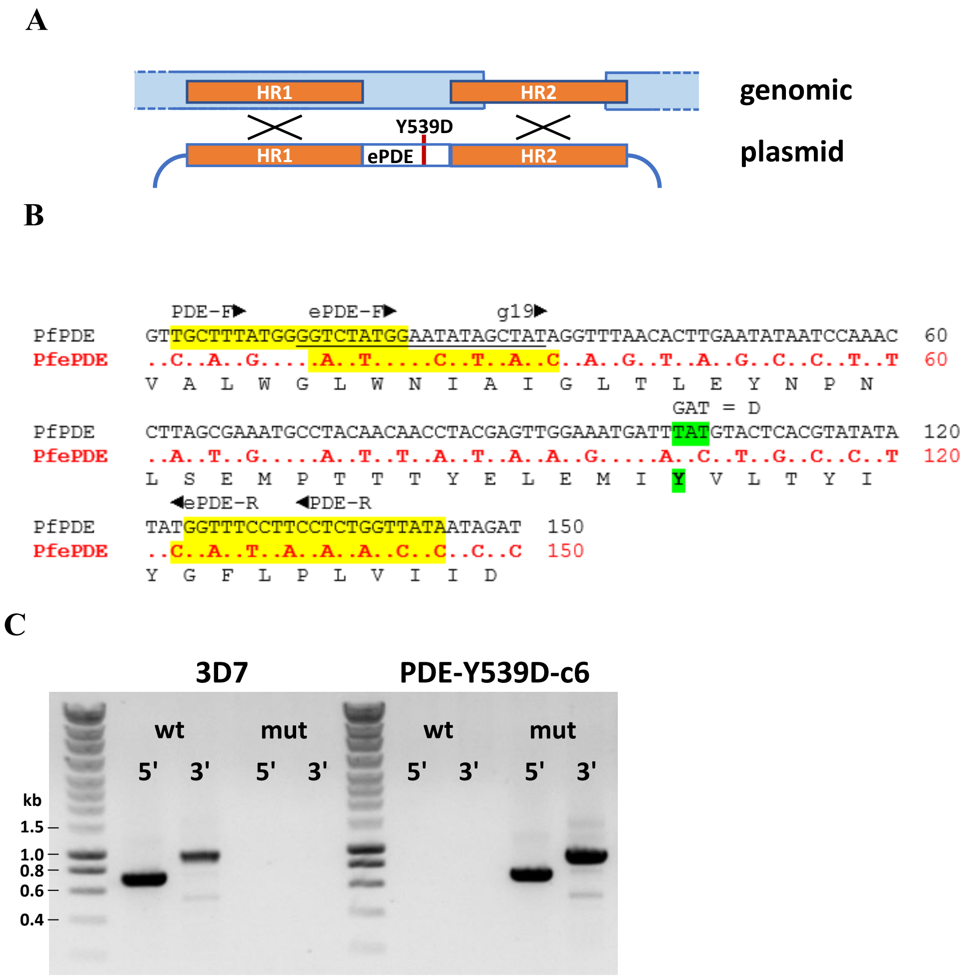

Fig. S4. Generation of transgenic parasites carrying a mutation in *Pf*PDEβ.
(**A**) Schematic representation of the very end of exon 1 where the Y539D mutation is located and the beginning of exon 2 (blue boxes) of the *Pfpdeβ* locus (above) and of the transfection constructs (below)**.** The homology regions (HR) for double homologous recombination and the position of the mutated residue Y539D are indicated. (**B**) Alignment of the *Pfpdeβ* wild type sequence (top) and the recodonised sequence (ePDE) in red (below). Dots indicate identical bases. The amino acid sequence is shown at the bottom. The mutated residue is highlighted in green and the modified codon the mutant shown above. The RNA guide sequence g19 is underlined. Primer sequences that were used in PCR to differentiate between wild type and transgenic parasites are indicated in yellow. (**C**) Genotyping of a transgenic parasite clone carrying a mutation in *Pf*PDEβ. Correct 5'- and 3' homologous recombination was detected by PCR on genomic DNA. For 5' integration primer Int-F in the sequence upstream of the HR1 region was used in combination with either the PDE-R (wild type) or ePDE-R (mutant) primer. For 3' integration primer Int-R in the sequence downstream of HR2 was used in combination with either PDE-F (wt) or ePDE-F (mut). SmartLadder (Eurogentec) was used as DNA molecular weight marker and relevant bands are indicated in kilobases (kb).

A

Strain	IC50 of CQ (nM)	
WT 3D7	11.4 ± 5.3	
	**R-parasites**	**GER-parasites**
*Pf*PKG - R420I	16.7 ± 7.2	12.0 ± 4.4
*Pf*PKG - H524N	17.5 ± 3.4	21.0 ± 8.7
*Pf*PKG - H524Y	12.3 ± 7.9	12.9 ± 3.2
*Pf*PKG - D597Y	12.2 ± 1.6	8.2 ± 3.9
*Pf*PDEβ-Y539D	ND	13.6 ± 0.5

B

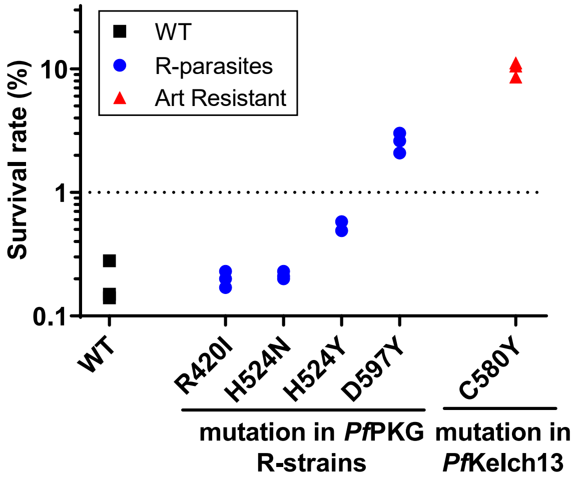

Fig. S5. Sensitivity of resistant parasites to antimalarial compounds. (A) Sensitivity of UA2239-resistant strains (R and GER-parasites) to chloroquine (CQ). Data are mean ± SD of 3 independent experiments performed in duplicates. ND: Not determined. **(B)** Sensitivity of R-parasites to dihydroartemisinin (DHA). Sensitivity was determined using ring-stage survival assay. A survival rate <1% indicates sensitive strains. 3D7 WT served as the sensitive control and the NF54 Kelch13 C580Y as resistant control. Data are from 3 independent experiments.

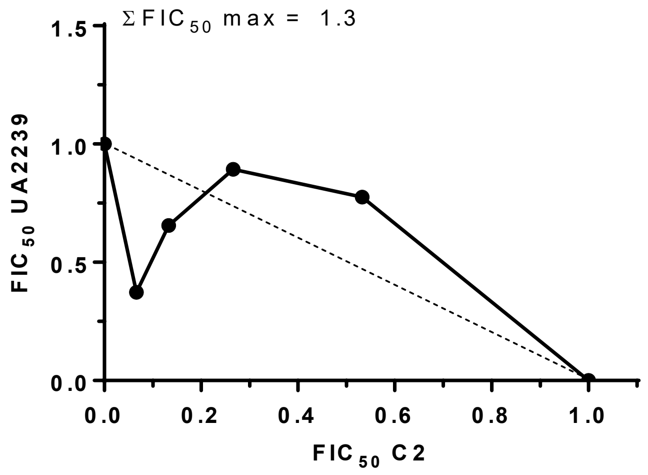

Fig. S6. Interaction between UA2239 and C2. *In vitro* interaction between UA2239 and C2 for their antimalarial activity is represented as an isobologram. The maximal sum of FICs (ΣFIC50 max) is indicated. Data presented are from one representative experiment (n=3 independent experiments).

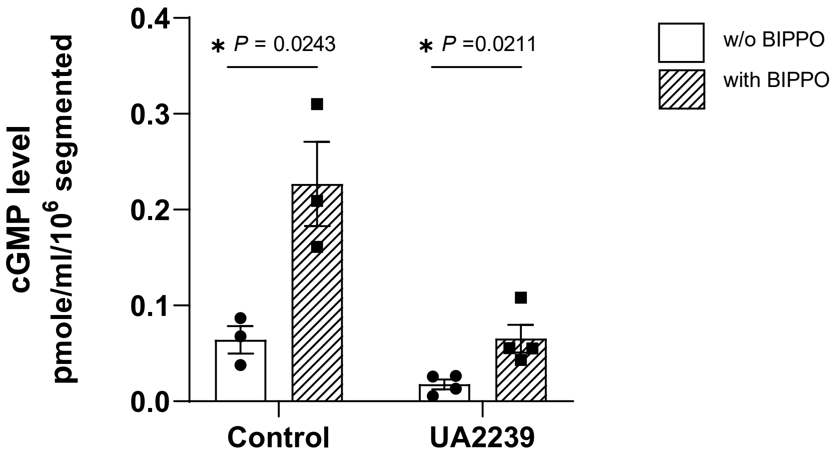

Fig. S7. Effect of the PDE inhibitor BIPPO on the intracellular cGMP levels in presence of UA2239. Purified schizonts (3D7) were allowed to develop in the presence of 1.5 µM C2 (to prevent egress) either without or with 750 nM UA2239. BIPPO was added to samples with C2 only or with C2 and 750 nM UA2239 at 42 hpi for 3 min (n=3 or 4 independent experiments, mean ± SEM). Two-tailed Student t test was used to calculate *P* value.

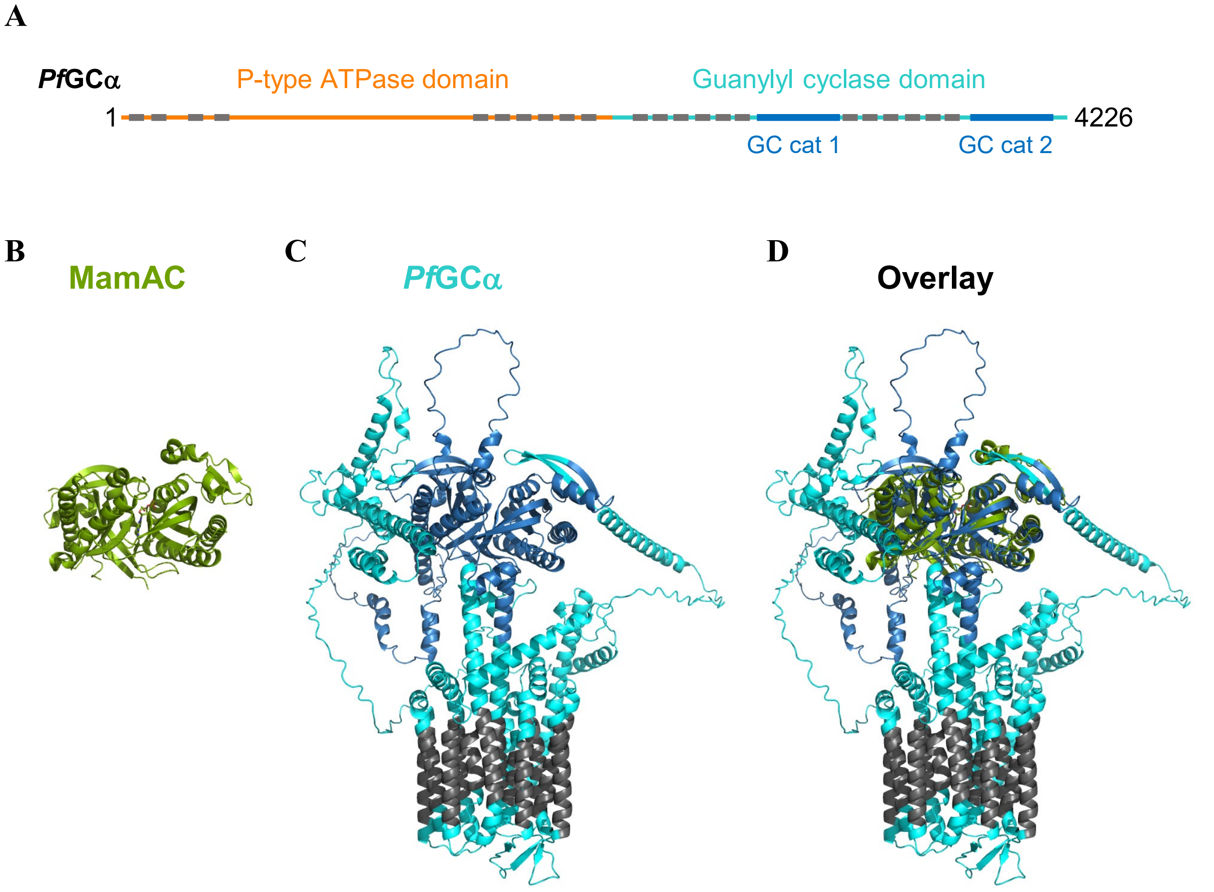

Fig. S8. Model of the 3D structure of *Pf*GCα. (**A**) Schematic representation of the full-length *Pf*GCα. The guanylyl cyclase domain (residues 2741-4226) is shown in cyan and the two catalytic sub-domains (residues 3007-3314 and 3963-4151) are in dark blue. The position of the transmembrane helices as predicted by DeepTMHMM (*77*) are shown with grey boxes. (**B**) Structure of the adenylyl cyclase domain of MamAC determined by X-ray diffraction at 2.50 Å (PBD 1CS4). Chains A and B, forming the adenylyl cyclase domain, are shown in green cartoons. (**C**) AlphaFold model of the guanylyl cyclase domain of *Pf*GCα shown in cartoons, using the same color code as in (A). (**D**) Overlay of MamAC and *Pf*GCα structures, aligned on the cyclase domains.

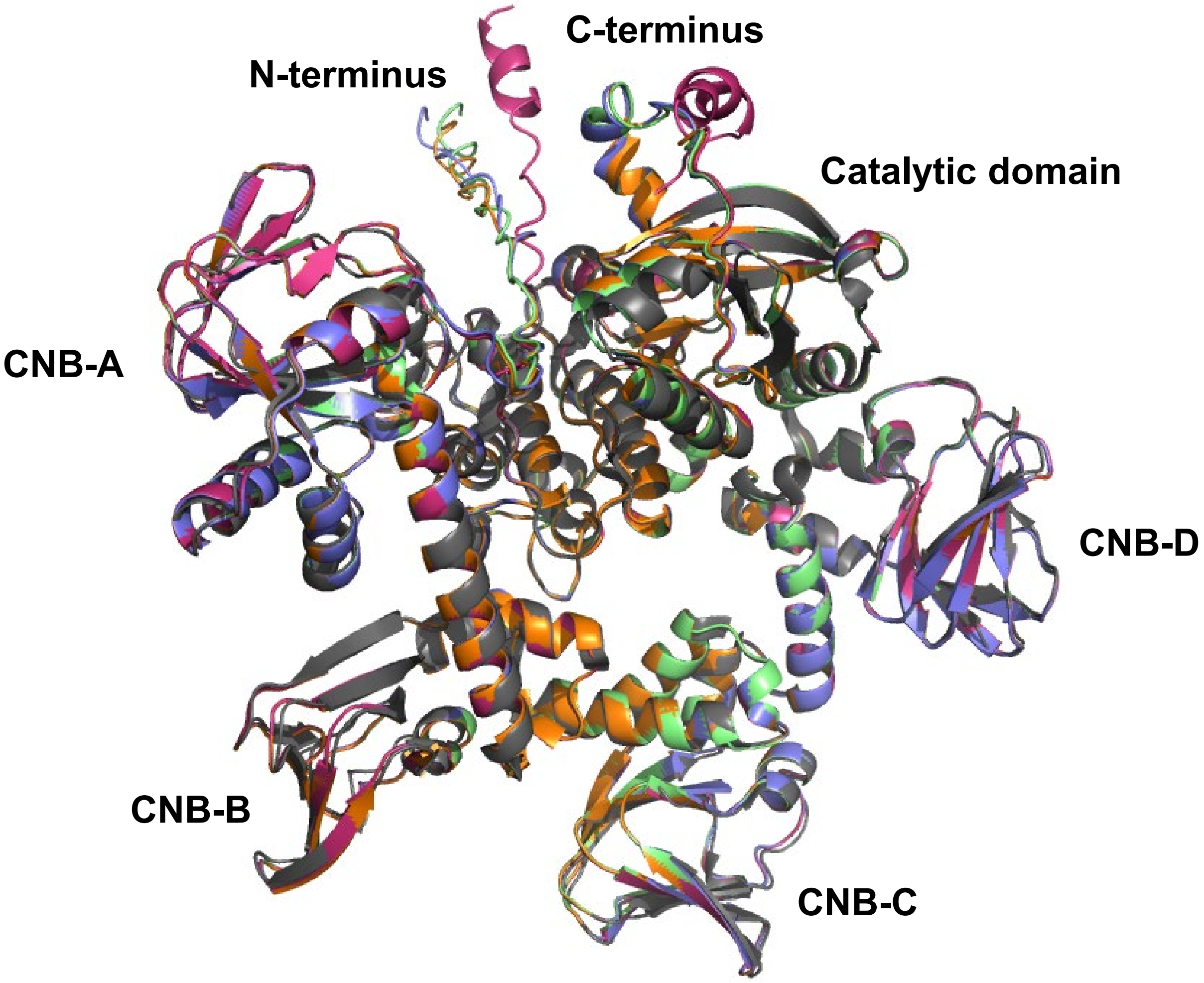

Fig. S9. Crystal structure of *Pf*PKG and AlphaFold models of *Pf*PKG mutants. The crystal structure of *Pf*PKG (grey) determined at 2.54 Å (PDB 8EM8) *(47)* is superimposed with the four structural models of the mutants generated by AlfaFold2 and the PDB 8EM8. Mutated *Pf*PKG are colored as follow: R420I in green, H524N in purple, H524Y in orange and D597Y in pink. The pairwise rmsd values vary between 0.456 and 0.475 Å over 771 amino-acids. The only differences are observed at the N-terminal (residues 1-27) and C-terminal (residues 818-853) regions, which are predicted to be poorly structured. The different domains are indicated on the figure.

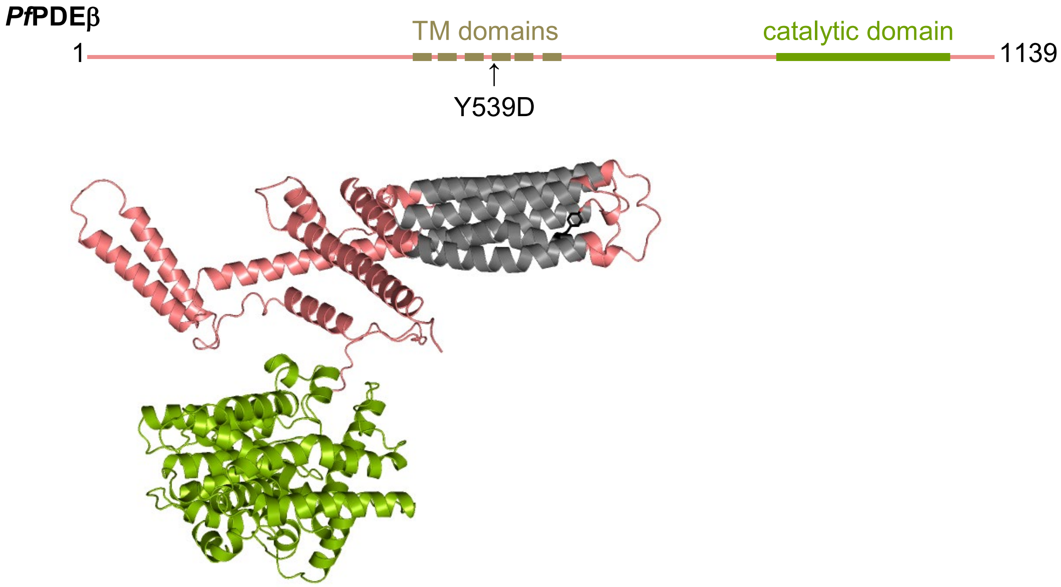

Fig. S10. Schematic representation and AlphaFold model of *Pf*PDEβ (PDB computed structure: AF-Q8I6Z7-F1-v4). The catalytic domain is shown in green and the 6 predicted transmembrane α-helices are in grey. The Y539 located in the 4th helix that is mutated in the resistant parasite line is in black. The structural model shows amino acid 342 to 1139.

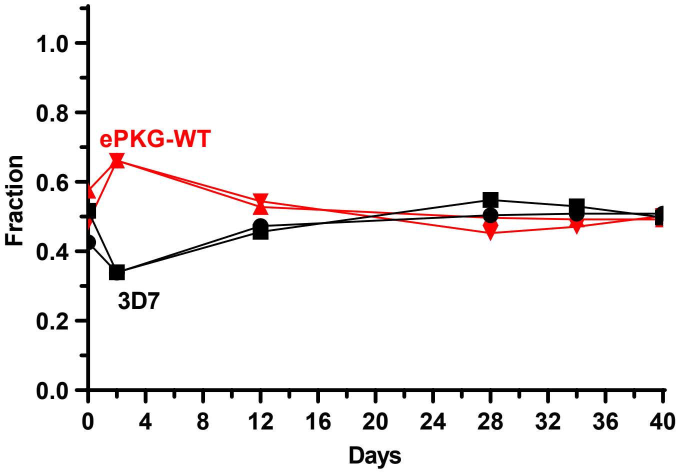

Fig. S11. Pairwise competitive growth assay followed by qPCR between ePKG-WT and 3D7 WT strains. Cultures for the *Pf*PKG competition assays were started at approximately equal parasitemia for both strains, equivalent to a fraction of around 0.5. The fraction remains at 0.5 if both strains grow at the same rate. The fraction rises to 1 if one strain grows faster than the other and finally represents 100% of the parasites (n=2).

Treatment	Number of segmented schizonts analyzed	% of intact PVM
Untreated	72	90.3%
UA2239 from 6 hpi	116	88.8%
UA2239 from 36 hpi	195	98.0%
C2 from 36 hpi	139	82.0%

Table S1. Percentage of parasites whose PVM did not rupture after treatment. iRBCs were treated with 2 μM UA2239 starting at 6 hpi or 36 hpi or with 1.5 μM C2 starting at 36 hpi. Parasites were processed for analysis by electron microcopy at 44-46 hpi.

REFERENCES AND NOTES

1. WHO, World malaria report 2024. https://who.int/teams/global-malaria-programme/reports/world-malaria-report-2024.

2. M. S. Datoo, A. Dicko, H. Tinto, J.-B. Ouédraogo, M. Hamaluba, A. Olotu, E. Beaumont, F. Ramos Lopez, H. M. Natama, S. Weston, M. Chemba, Y. D. Compaore, D. Issiaka, D. Salou, A. M. Some, S. Omenda, A. Lawrie, P. Bejon, H. Rao, D. Chandramohan, R. Roberts, S. Bharati, L. Stockdale, S. Gairola, B. M. Greenwood, K. J. Ewer, J. Bradley, P. S. Kulkarni, U. Shaligram, A. V. S. Hill, R21/Matrix-M Phase 3 Trial Group, Safety and efficacy of malaria vaccine candidate R21/Matrix-M in African children: A multicentre, double-blind, randomised, phase 3 trial. *Lancet* **403**, 533–544 (2024).

3. "Global Observatory on Health R&D" (World Health Organization, 2024); https://who.int/observatories/global-observatory-on-health-research-and-development.

4. P. E. Duffy, J. P. Gorres, S. A. Healy, M. Fried, Malaria vaccines: A new era of prevention and control. *Nat. Rev. Microbiol.* **22**, 756–772 (2024).

5. H. Noedl, Y. Se, K. Schaecher, B. L. Smith, D. Socheat, M. M. Fukuda, Evidence of artemisinin-resistant malaria in western Cambodia. *N. Engl. J. Med.* **359**, 2619–2620 (2008).

6. J. N. Burrows, S. Duparc, W. E. Gutteridge, R. Hooft Van Huijsduijnen, W. Kaszubska, F. Macintyre, S. Mazzuri, J. J. Möhrle, T. N. C. Wells, New developments in anti-malarial target candidate and product profiles. *Malar. J.* **16**, 26 (2017).

7. A. H. Lee, L. S. Symington, D. A. Fidock, DNA repair mechanisms and their biological roles in the malaria parasite *Plasmodium falciparum*. *Microbiol. Mol. Biol. Rev.* **78**, 469–486 (2014).

8. Z. Liu, J. Miao, L. Cui, Gametocytogenesis in malaria parasite: Commitment, development and regulation. *Future Microbiol.* **6**, 1351–1369 (2011).

9. T. Cheviet, S. Wein, G. Bourchenin, M. Lagacherie, C. Périgaud, R. Cerdan, S. Peyrottes, β-Hydroxy- and β-aminophosphonate acyclonucleosides as potent inhibitors of *Plasmodium falciparum* growth. *J. Med. Chem.* **63**, 8069–8087 (2020).

10. L. P. Jordheim, D. Durantel, F. Zoulim, C. Dumontet, Advances in the development of nucleoside and nucleotide analogues for cancer and viral diseases. *Nat. Rev. Drug Discov.* **12**, 447–464 (2013).

11. K. L. Seley-Radtke, M. K. Yates, The evolution of nucleoside analogue antivirals: A review for chemists and non-chemists. Part 1: Early structural modifications to the nucleoside scaffold. *Antiviral Res.* **154**, 66–86 (2018).

12. M. S. Y. Tan, M. J. Blackman, Malaria parasite egress at a glance. *J. Cell Sci.* **134**, jcs257345 (2021).

13. J. D. Dvorin, D. E. Goldberg, *Plasmodium* egress across the parasite life cycle. *Annu. Rev. Microbiol.* **76**, 67–90 (2022).

14. C. R. Collins, F. Hackett, M. Strath, M. Penzo, C. Withers-Martinez, D. A. Baker, M. J. Blackman, Malaria parasite cGMP-dependent protein kinase regulates blood stage merozoite secretory organelle discharge and egress. *PLOS Pathog.* **9**, e1003344 (2013).

15. A. C. Balestra, K. Koussis, N. Klages, S. A. Howell, H. R. Flynn, M. Bantscheff, C. Pasquarello, A. J. Perrin, L. Brusini, P. Arboit, O. Sanz, L. P.-B. Castaño, C. Withers-Martinez, A. Hainard, S. Ghidelli-Disse, A. P. Snijders, D. A. Baker, M. J. Blackman, M. Brochet, Ca^{2+} signals critical for egress and gametogenesis in malaria parasites depend on a multipass membrane protein that interacts with PKG. *Sci. Adv.* **7**, eabe5396 (2021).

16. D. A. Baker, L. G. Drought, C. Flueck, S. D. Nofal, A. Patel, M. Penzo, E. M. Walker, Cyclic nucleotide signalling in malaria parasites. *Open Biol.* **7**, 170213 (2017).

17. S. D. Nofal, A. Patel, M. J. Blackman, C. Flueck, D. A. Baker, *Plasmodium falciparum* guanylyl cyclase-alpha and the activity of its appended P4-ATPase domain are essential for cGMP synthesis and blood-stage egress. *mBio* **12**, e02694-20 (2021).

18. W. J. Moss, L. Brusini, R. Kuehnel, M. Brochet, K. M. Brown, Apicomplexan phosphodiesterases in cyclic nucleotide turnover: Conservation, function, and therapeutic potential. *mBio* **15**, e03056-23 (2024).

19. R. G. K. Donald, T. Zhong, H. Wiersma, B. Nare, D. Yao, A. Lee, J. Allocco, P. A. Liberator, Anticoccidial kinase inhibitors: Identification of protein kinase targets secondary to cGMP-dependent protein kinase. *Mol. Biochem. Parasitol.* **149**, 86–98 (2006).

20. D. A. Baker, A. N. Matralis, S. A. Osborne, J. M. Large, M. Penzo, Targeting the malaria parasite cGMP-dependent protein kinase to develop new drugs. *Front. Microbiol.* **11**, 602803 (2020).

21. D. A. Baker, L. B. Stewart, J. M. Large, P. W. Bowyer, K. H. Ansell, M. B. Jiménez-Díaz, M. E. Bakkouri, K. Birchall, K. J. Dechering, N. S. Bouloc, P. J. Coombs, D. Whalley, D. J. Harding, E. Smiljanic-Hurley, M. C. Wheldon, E. M. Walker, J. T. Dessens, M. J. Lafuente, L. M. Sanz, F.-J. Gamo, S. B. Ferrer, R. Hui, T. Bousema, I. Angulo-Barturén, A. T. Merritt, S. L. Croft, W. E. Gutteridge, C. A. Kettleborough, S. A. Osborne, A potent series targeting the malarial cGMP-dependent protein kinase clears infection and blocks transmission. *Nat. Commun.* **8**, 430 (2017).

22. M. Vanaerschot, J. M. Murithi, C. F. A. Pasaje, S. Ghidelli-Disse, L. Dwomoh, M. Bird, N. Spottiswoode, N. Mittal, L. B. Arendse, E. S. Owen, K. J. Wicht, G. Siciliano, M. Bösche, T. Yeo, T. R. S. Kumar, S. Mok, E. F. Carpenter, M. J. Giddins, O. Sanz, S. Ottilie, P. Alano, K. Chibale, M. Llinás, A.-C. Uhlemann, M. Delves, A. B. Tobin, C. Doerig, E. A. Winzeler, M. C. S. Lee, J. C. Niles, D. A. Fidock, Inhibition of resistance-refractory *P. falciparum* kinase PKG delivers prophylactic, blood stage, and transmission-blocking antiplasmodial activity. *Cell Chem. Biol.* **27**, 806–816.e8 (2020).

23. L. Wentzinger, S. Bopp, H. Tenor, J. Klar, R. Brun, H. P. Beck, T. Seebeck, Cyclic nucleotide-specific phosphodiesterases of *Plasmodium falciparum*: *Pf*PDEα, a non-essential cGMP-specific PDE that is an integral membrane protein. *Int. J. Parasitol.* **38**, 1625–1637 (2008).

24. B. L. Howard, K. L. Harvey, R. J. Stewart, M. F. Azevedo, B. S. Crabb, I. G. Jennings, P. R. Sanders, D. T. Manallack, P. E. Thompson, C. J. Tonkin, P. R. Gilson, Identification of potent phosphodiesterase inhibitors that demonstrate cyclic cucleotide-dependent functions in apicomplexan parasites. *ACS Chem. Biol.* **10**, 1145–1154 (2015).

25. C. Flueck, L. G. Drought, A. Jones, A. Patel, A. J. Perrin, E. M. Walker, S. D. Nofal, A. P. Snijders, M. J. Blackman, D. A. Baker, Phosphodiesterase beta is the master regulator of cAMP signalling during malaria parasite invasion. *PLOS Biol.* **17**, e3000154 (2019).

26. P.-J. Gomez-Gonzalez, A. Gupta, L. G. Drought, A. Patel, J. Okombo, M. van der Watt, R. Walker-Gray, K. A. Schindler, A. Y. Burkhard, T. Yeo, S. K. Narwal, T. S. Bloxham, C. Flueck, E. M. Walker, J. A. Rey, K. J. Fairhurst, J. Reader, H. Park, H. G. Pollard, L. B. Stewart, L. Brandner-Garrod, M. Kristan, G.-J. Sterk, Y. M. van Nuland, E. Manko, D. A. van Schalkwyk, Y. Zheng, R. Leurs, K. J. Dechering, A. C. C. Aguiar, R. V. C. Guido, D. B. Pereira, P. K. Tumwebaze, S. L. Nosbya, P. J. Rosenthal, R. A. Cooper, M. Palmer, T. Parkinson, J. N. Burrows, A.-C. Uhlemann, L.-M. Birkholtz, J. L. Small-Saunders, J. Duffy, D. A. Fidock, A. Brown, M. Gardner, D. A. Baker, Inhibitors of malaria parasite cyclic nucleotide phosphodiesterases block asexual blood-stage development and mosquito transmission. *Sci. Adv.* **10**, eadq1383 (2024).

27. R. W. Moon, C. J. Taylor, C. Bex, R. Schepers, D. Goulding, C. J. Janse, A. P. Waters, D. A. Baker, O. Billker, A cyclic GMP signalling module that regulates gliding motility in a malaria parasite. *PLOS Pathog.* **5**, e1000599 (2009).

28. H. Ginsburg, W. D. Stein, The new permeability pathways induced by the malaria parasite in the membrane of the infected erythrocyte: Comparison of results using different experimental techniques. *J. Membr. Biol.* **197**, 113–134 (2004).

29. K. Kirk, H. A. Horner, B. C. Elford, J. C. Ellory, C. I. Newbold, Transport of diverse substrates into malaria-infected erythrocytes via a pathway showing functional characteristics of a chloride channel. *J. Biol. Chem.* **269**, 3339–3347 (1994).

30. R. Huang, L. Pei, Q. Liu, S. Chen, H. Dou, G. Shu, Z. Yuan, J. Lin, G. Peng, W. Zhang, H. Fu, Isobologram analysis: A comprehensive review of methodology and current research. *Front. Pharmacol.* **10**, 1222 (2019).

31. T. Cheviet, I. Lefebvre-Tournier, S. Wein, S. Peyrottes, *Plasmodium* purine metabolism and its inhibition by nucleoside and nucleotide analogues. *J. Med. Chem.* **62**, 8365–8391 (2019).

32. J. de Jersey, A. Holý, D. Hocková, L. Naesens, D. T. Keough, L. W. Guddat, 6-Oxopurine phosphoribosyltransferase: A target for the development of antimalarial drugs. *Curr. Top. Med. Chem.* **11**, 2085–2102 (2011).

33. D. T. Keough, D. Hocková, Z. Janeba, T.-H. Wang, L. Naesens, M. D. Edstein, M. Chavchich, L. W. Guddat, Aza-acyclic nucleoside phosphonates containing a second phosphonate croup as inhibitors of the human, *Plasmodium falciparum* and *vivax* 6-oxopurine phosphoribosyltransferases and their prodrugs as antimalarial agents. *J. Med. Chem.* **58**, 827–846 (2015).

34. L. Eyer, R. Nencka, E. de Clercq, K. Seley-Radtke, D. Růžek, Nucleoside analogs as a rich source of antiviral agents active against arthropod-borne flaviviruses. *Antivir. Chem. Chemother.* **26**, 2040206618761299 (2018).

35. T. Chu, K. Lingelbach, J. M. Przyborski, Genetic evidence strongly support an essential role for PfPV1 in intra-erythrocytic growth of *P. falciparum*. *PLOS ONE* **6**, e18396 (2011).

36. M. E. Wickham, J. G. Culvenor, A. F. Cowman, Selective inhibition of a two-step egress of malaria parasites from the host erythrocyte. *J. Biol. Chem.* **278**, 37658–37663 (2003).

37. P. Suaréz-Cortés, F. Silvestrini, P. Alano, A fast, non-invasive, quantitative staining protocol provides insights in *Plasmodium falciparum* gamete egress and in the role of osmiophilic bodies. *Malar. J.* **13**, 389 (2014).

38. G. Bouyer, D. Barbieri, F. Dupuy, A. Marteau, A. Sissoko, M.-E. N'Dri, G. Neveu, L. Bedault, N. Khodabux, D. Roman, S. Houzé, G. Siciliano, P. Alano, R. M. Martins, J.-J. Lopez-Rubio, J.

Clain, R. Duval, S. Egée, C. Lavazec, *Plasmodium falciparum* sexual parasites regulate infected erythrocyte permeability. *Commun. Biol.* **3**, 726 (2020).

39. M. El Bakkouri, I. Kouidmi, A. K. Wernimont, M. Amani, A. Hutchinson, P. Loppnau, J. J. Kim, C. Flueck, J. R. Walker, A. Seitova, G. Senisterra, Y. Kakihara, C. Kim, M. J. Blackman, C. Calmettes, D. A. Baker, R. Hui, Structures of the cGMP-dependent protein kinase in malaria parasites reveal a unique structural relay mechanism for activation. *Proc. Natl. Acad. Sci. U.S.A.* **116**, 14164–14173 (2019).

40. J. J. Kim, C. Flueck, E. Franz, E. Sanabria-Figueroa, E. Thompson, R. Lorenz, D. Bertinetti, D. A. Baker, F. W. Herberg, C. Kim, Crystal structures of the carboxyl cGMP binding domain of the *Plasmodium falciparum* cGMP-dependent protein kinase reveal a novel capping triad crucial for Merozoite egress. *PLOS Pathog.* **11**, e1004639 (2015).

41. S. Eksi, B. J. Morahan, Y. Haile, T. Furuya, H. Jiang, O. Ali, H. Xu, K. Kiattibutr, A. Suri, B. Czesny, A. Adeyemo, T. G. Myers, J. Sattabongkot, X. Su, K. C. Williamson, *Plasmodium falciparum* gametocyte development 1 (Pfgdv1) and gametocytogenesis early gene identification and commitment to sexual development. *PLOS Pathog.* **8**, e1002964 (2012).

42. M. Filarsky, S. A. Fraschka, I. Niederwieser, N. M. B. Brancucci, E. Carrington, E. Carrió, S. Moes, P. Jenoe, R. Bártfai, T. S. Voss, GDV1 induces sexual commitment of malaria parasites by antagonizing HP1-dependent gene silencing. *Science* **359**, 1259–1263 (2018).

43. L. McRobert, C. J. Taylor, W. Deng, Q. L. Fivelman, R. M. Cummings, S. D. Polley, O. Billker, D. A. Baker, Gametogenesis in malaria parasites is mediated by the cGMP-dependent protein kinase. *PLOS Biol.* **6**, e139 (2008).

44. J. A. Byun, K. Van, J. Huang, P. Henning, E. Franz, M. Akimoto, F. W. Herberg, C. Kim, G. Melacini, Mechanism of allosteric inhibition in the *Plasmodium falciparum* cGMP-dependent protein kinase. *J. Biol. Chem.* **295**, 8480–8491 (2020).

45. J. Jumper, R. Evans, A. Pritzel, T. Green, M. Figurnov, O. Ronneberger, K. Tunyasuvunakool, R. Bates, A. Žídek, A. Potapenko, A. Bridgland, C. Meyer, S. A. A. Kohl, A. J. Ballard, A.

Cowie, B. Romera-Paredes, S. Nikolov, R. Jain, J. Adler, T. Back, S. Petersen, D. Reiman, E. Clancy, M. Zielinski, M. Steinegger, M. Pacholska, T. Berghammer, S. Bodenstein, D. Silver, O. Vinyals, A. W. Senior, K. Kavukcuoglu, P. Kohli, D. Hassabis, Highly accurate protein structure prediction with AlphaFold. *Nature* **596**, 583–589 (2021).

46. D. A. Baker, Adenylyl and guanylyl cyclases from the malaria parasite *Plasmodium falciparum*. *IUBMB Life* **56**, 535–540 (2004).

47. J. A. Gilleran, K. Ashraf, M. Delvillar, T. Eck, R. Fondekar, E. B. Miller, A. Hutchinson, A. Dong, A. Seitova, M. L. De Souza, D. Augeri, L. Halabelian, J. Siekierka, D. P. Rotella, J. Gordon, W. E. Childers, M. C. Grier, B. L. Staker, J. Y. Roberge, P. Bhanot, Structure-activity relationship of a pyrrole based series of *Pf*PKG inhibitors as anti-malarials. *J. Med. Chem.* **67**, 3467–3503 (2024).

48. Y. Zhang, Y. Gao, X. Wen, H. Ma, Current prodrug strategies for improving oral absorption of nucleoside analogues. *Asian J. Pharm. Sci.* **9**, 65–74 (2014).

49. K. Seley-Radtke, J. Deval, Advances in antiviral nucleoside analogues and their prodrugs. *Antivir. Chem. Chemother.* **26**, 2040206618781410 (2018).

50. E. Groaz, S. De Jonghe, Overview of biologically active nucleoside phosphonates. *Front. Chem.* **8**, 616863 (2021).

51. G. Zhang, Y. Liu, A. E. Ruoho, J. H. Hurley, Structure of the adenylyl cyclase catalytic core. *Nature* **386**, 247–253 (1997).

52. P. J. Artymiuk, A. R. Poirrette, D. W. Rice, P. Willett, A polymerase I palm in adenylyl cyclase? *Nature* **388**, 33–34 (1997).

53. D. J. Carucci, A. A. Witney, D. K. Muhia, D. C. Warhurst, P. Schaap, M. Meima, J.-L. Li, M. C. Taylor, J. M. Kelly, D. A. Baker, Guanylyl cyclase activity associated with putative bifunctional integral membrane proteins in *Plasmodium falciparum*. *J. Biol. Chem.* **275**, 22147–22156 (2000).

54. A. Patel, S. D. Nofal, M. J. Blackman, D. A. Baker, CDC50 orthologues in *Plasmodium falciparum* have distinct roles in merozoite egress and trophozoite maturation. *mBio* **13**, e0163522 (2022).

55. R. M. Kuehnel, E. Ganga, A. C. Balestra, C. Suarez, M. Wyss, N. Klages, L. Brusini, B. Maco, N. Brancucci, T. S. Voss, D. Soldati, M. Brochet, A *Plasmodium* membrane receptor platform integrates cues for egress and invasion in blood forms and activation of transmission stages. *Sci. Adv.* **9**, eadf2161 (2023).

56. A. S. Paul, A. Miliu, J. A. Paulo, J. M. Goldberg, A. M. Bonilla, L. Berry, M. Seveno, C. Braun-Breton, A. L. Kosber, B. Elsworth, J. S. N. Arriola, M. Lebrun, S. P. Gygi, M. H. Lamarque, M. T. Duraisingh, Co-option of *Plasmodium falciparum* PP1 for egress from host erythrocytes. *Nat. Commun.* **11**, 3532 (2020).

57. M. Seveno, M. N. Loubens, L. Berry, A. Graindorge, M. Lebrun, C. Lavazec, M. H. Lamarque, The malaria parasite PP1 phosphatase controls the initiation of the egress pathway of asexual blood-stages by regulating the rounding-up of the vacuole. *PLOS Pathog.* **21**, e1012455 (2025).

58. C. Lee, E. S. Ünlü, N. F. D. White, J. Almagro-Garcia, C. Ariani, R. D. Pearson, Pf-HaploAtlas: An interactive web app for spatiotemporal analysis of *P. falciparum* genes. bioRxiv 603783 [Preprint] (2024). https://doi.org/10.1101/2024.07.16.603783.

59. E. Franz, M. J. Knape, F. W. Herberg, cGMP binding domain D mediates a unique activation mechanism in *Plasmodium falciparum* PKG. *ACS Infect. Dis.* **4**, 415–423 (2018).

60. R. E. Desjardins, C. J. Canfield, J. D. Haynes, J. D. Chulay, Quantitative assessment of antimalarial activity in vitro by a semiautomated microdilution technique. *Antimicrob. Agents Chemother.* **16**, 710–718 (1979).

61. M. L. Ancelin, M. Calas, V. Vidal-Sailhan, S. Herbute, P. Ringwald, H. J. Vial, Potent inhibitors of *Plasmodium* phospholipid metabolism with a broad spectrum of in vitro antimalarial activities. *Antimicrob. Agents Chemother.* **47**, 2590–2597 (2003).

62. M. C. Berenbaum, A method for testing for synergy with any number of agents. *J. Infect. Dis.* **137**, 122–130 (1978).

63. S. Gupta, M. M. Thapar, W. H. Wernsdorfer, A. Bjorkman, In vitro interactions of artemisinin with atovaquone, quinine, and mefloquine against *Plasmodium falciparum*. *Antimicrob. Agents Chemother.* **46**, 1510–1515 (2002).

64. C. Lavazec, S. Sanyal, T. J. Templeton, Expression switching in the stevor and Pfmc-2TM superfamilies in *Plasmodium falciparum*. *Mol. Microbiol.* **64**, 1621–1634 (2007).

65. H. Li, R. Durbin, Fast and accurate short read alignment with Burrows–Wheeler transform. *Bioinformatics* **25**, 1754–1760 (2009).

66. A. M. Bolger, M. Lohse, B. Usadel, Trimmomatic: A flexible trimmer for Illumina sequence data. *Bioinformatics* **30**, 2114–2120 (2014).

67. A. McKenna, M. Hanna, E. Banks, A. Sivachenko, K. Cibulskis, A. Kernytsky, K. Garimella, D. Altshuler, S. Gabriel, M. Daly, M. A. DePristo, The Genome Analysis Toolkit: A MapReduce framework for analyzing next-generation DNA sequencing data. *Genome Res.* **20**, 1297–1303 (2010).

68. T. Rausch, T. Zichner, A. Schlattl, A. M. Stütz, V. Benes, J. O. Korbel, DELLY: Structural variant discovery by integrated paired-end and split-read analysis. *Bioinformatics* **28**, i333–i339 (2012).

69. MalariaGEN, M. M. Abdel Hamid, M. H. Abdelraheem, D. O. Acheampong, A. Ahouidi, M. Ali, J. Almagro-Garcia, A. Amambua-Ngwa, C. Amaratunga, L. Amenga-Etego, B. Andagalu, T. Anderson, V. Andrianaranjaka, I. Aniebo, E. Aninagyei, F. Ansah, P. O. Ansah, T. Apinjoh, P. Arnaldo, E. Ashley, S. Auburn, G. A. Awandare, H. Ba, V. Baraka, A. Barry, P. Bejon, G. I. Bertin, M. F. Boni, S. Borrmann, T. Bousema, M. Bouyou-Akotet, O. Branch, P. C. Bull, H. Cheah, K. Chindavongsa, T. Chookajorn, K. Chotivanich, A. Claessens, D. J. Conway, V. Corredor, E. Courtier, A. Craig, U. D'Alessandro, S. Dama, N. Day, B. Denis, M. Dhorda, M. Diakite, A. Djimde, C. Dolecek, A. Dondorp, S. Doumbia, C. Drakeley, E. Drury, P. Duffy, D.

F. Echeverry, T. G. Egwang, S. M. M. Enosse, B. Erko, R. M. Fairhurst, A. Faiz, C. A. Fanello, M. Fleharty, M. Forbes, M. Fukuda, D. Gamboa, A. Ghansah, L. Golassa, S. Goncalves, G. L. A. Harrison, S. A. Healy, J. A. Hendry, A. Hernandez-Koutoucheva, T. T. Hien, C. A. Hill, F. Hombhanje, A. Hott, Y. Htut, M. Hussein, M. Imwong, D. Ishengoma, S. A. Jackson, C. G. Jacob, J. Jeans, K. J. Johnson, C. Kamaliddin, E. Kamau, J. Keatley, T. Kochakarn, D. S. Konate, A. Konaté, A. Kone, D. P. Kwiatkowski, M. P. Kyaw, D. Kyle, M. Lawniczak, S. K. Lee, M. Lemnge, P. Lim, C. Lon, K. M. Loua, C. I. Mandara, J. Marfurt, K. Marsh, R. J. Maude, M. Mayxay, O. Maïga-Ascofaré, O. Miotto, T. Mita, V. Mobegi, A. O. Mohamed, O. A. Mokuolu, J. Montgomery, C. M. Morang'a, I. Mueller, K. Murie, P. N. Newton, T. Ngo Duc, T. Nguyen, T.-N. Nguyen, T. Nguyen Thi Kim, H. Nguyen Van, H. Noedl, F. Nosten, R. Noviyanti, V. N.-N. Ntui, A. Nzila, L. I. Ochola-Oyier, H. Ocholla, A. Oduro, I. Omedo, M. A. Onyamboko, J.-B. Ouedraogo, K. Oyebola, W. A. Oyibo, R. Pearson, N. Peshu, A. P. Phyo, C. V. Plowe, R. N. Price, S. Pukrittayakamee, H. H. Quang, M. Randrianarivelojosia, J. C. Rayner, P. Ringwald, A. Rosanas-Urgell, E. Rovira-Vallbona, V. Ruano-Rubio, L. Ruiz, D. Saunders, A. Shayo, P. Siba, V. J. Simpson, M. S. Sissoko, C. Smith, X.-Z. Su, C. Sutherland, S. Takala-Harrison, A. Talman, L. Tavul, N. V. Thanh, V. Thathy, A. M. Thu, M. Toure, A. Tshefu, F. Verra, J. Vinetz, T. E. Wellems, J. Wendler, N. J. White, G. Whitton, W. Yavo, R. W. van der Pluijm, Pf7: An open dataset of *Plasmodium falciparum* genome variation in 20,000 worldwide samples. *Wellcome Open Res.* **8**, 22 (2023).

70. K. Labun, T. G. Montague, M. Krause, Y. N. Torres Cleuren, H. Tjeldnes, E. Valen, CHOPCHOP v3: Expanding the CRISPR web toolbox beyond genome editing. *Nucleic Acids Res.* **47**, W171–W174 (2019).

71. E. Knuepfer, M. Napiorkowska, C. van Ooij, A. A. Holder, Generating conditional gene knockouts in Plasmodium—A toolkit to produce stable DiCre recombinase-expressing parasite lines using CRISPR/Cas9. *Sci. Rep.* **7**, 3881 (2017).

72. D. A. Fidock, T. E. Wellems, Transformation with human dihydrofolate reductase renders malaria parasites insensitive to WR99210 but does not affect the intrinsic activity of proguanil. *Proc. Natl. Acad. Sci. U.S.A.* **94**, 10931–10936 (1997).

73. B. Witkowski, C. Amaratunga, N. Khim, S. Sreng, P. Chim, S. Kim, P. Lim, S. Mao, C. Sopha, B. Sam, J. M. Anderson, S. Duong, C. M. Chuor, W. R. Taylor, S. Suon, O. Mercereau-Puijalon, R. M. Fairhurst, D. Menard, Novel phenotypic assays for the detection of artemisinin-resistant *Plasmodium falciparum* malaria in Cambodia: In-vitro and ex-vivo drug-response studies. *Lancet Infect. Dis.* **13**, 1043–1049 (2013).

74. M. Ghorbal, M. Gorman, C. R. Macpherson, R. M. Martins, A. Scherf, J.-J. Lopez-Rubio, Genome editing in the human malaria parasite *Plasmodium falciparum* using the CRISPR-Cas9 system. *Nat. Biotechnol.* **32**, 819–821 (2014).

75. O. Korb, T. Stützle, T. E. Exner, "PLANTS: Application of ant colony optimization to structure-based drug fesign" in *Ant Colony Optimization and Swarm Intelligence*, M. Dorigo, L. M. Gambardella, M. Birattari, A. Martinoli, R. Poli, T. Stützle, Eds. (Springer, 2006), pp. 247–258.

76. M. Duffey, B. Blasco, J. N. Burrows, T. N. C. Wells, D. A. Fidock, D. Leroy, Assessing risks of Plasmodium falciparum resistance to select next-generation antimalarials. *Trends Parasitol.* **37**, 709–721 (2021).

77. J. Hallgren, K. Tsirigos, M. D. Pedersen, J. J. Almagro Armenteros, P. Marcatili, H. Nielsen, A. Krogh, O. Winther, DeepTMHMM predicts alpha and beta transmembrane proteins using deep neural network. bioRxiv 487609 [Preprint] (2022). https://doi.org/10.1101/2022.04.08.487609.
